# Supplementary material for: Characterizing the Causal Pathway for Genetic Variants Associated with Neurological Phenotypes Using Human Brain-Derived Proteome Data
Source: Am J Hum Genet. 2020 May 14;106(6):885–92. doi: 10.1016/j.ajhg.2020.04.007 (PMC7273531; doi:10.1016/j.ajhg.2020.04.007)
Supplement: Document S1 — Figures S1–S13 and Table S1 [file mmc1.pdf]

The American Journal of Human Genetics, Volume *106*

Supplemental Data

Characterizing the Causal Pathway for Genetic Variants Associated with Neurological Phenotypes Using Human Brain-Derived Proteome Data

Nelson K. Kibinge, Caroline L. Relton, Tom R. Gaunt, and Tom G. Richardson

Supplementary Figure 1 – A flowchart of the analysis pipeline for this study and key results

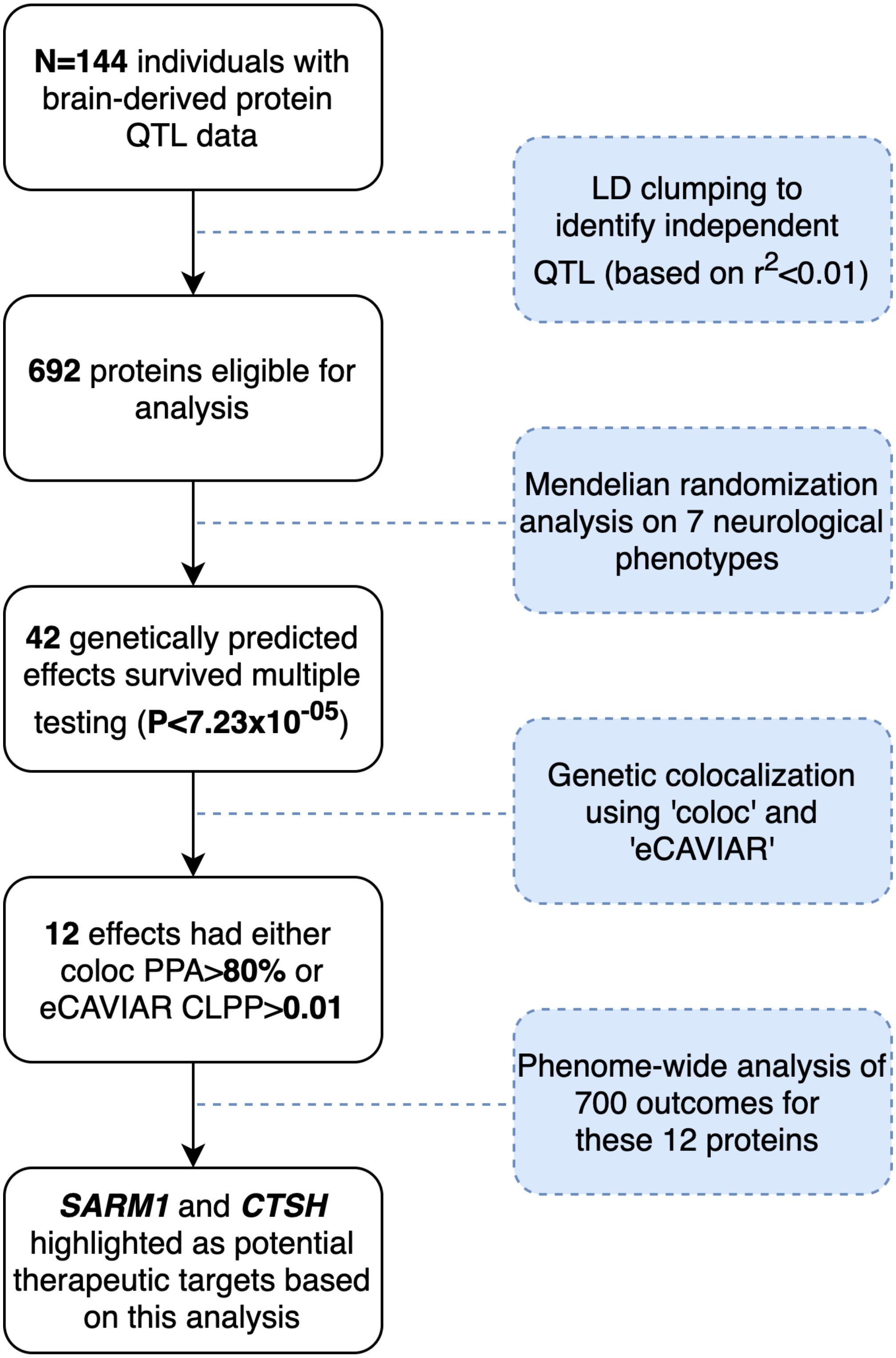

Supplementary Figure 2 – Gene expression profile for *ABCB9* across 54 tissue types in the GTEx project

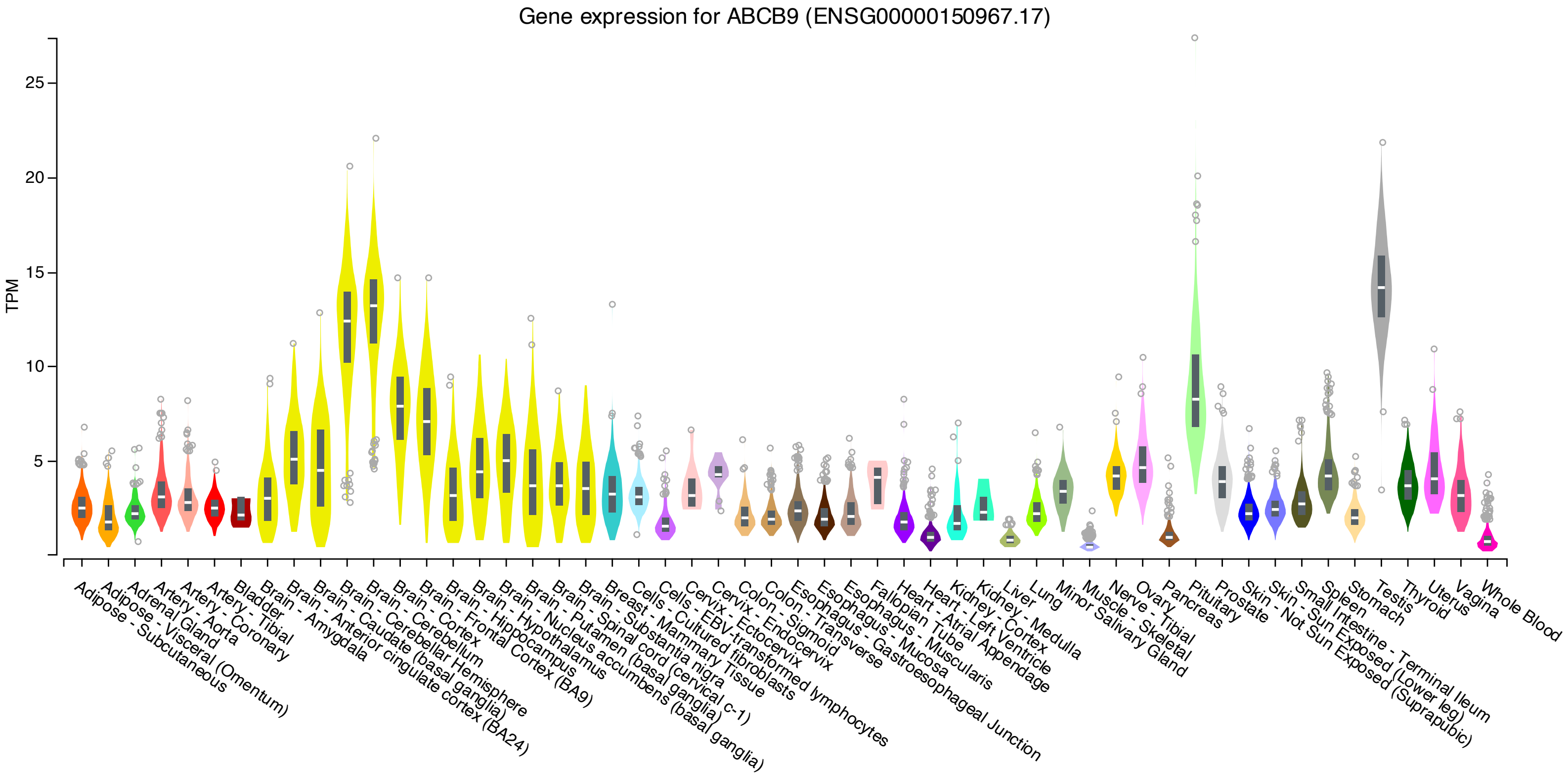

Supplementary Figure 3 – Gene expression profile for *CTSH* across 54 tissue types in the GTEx project

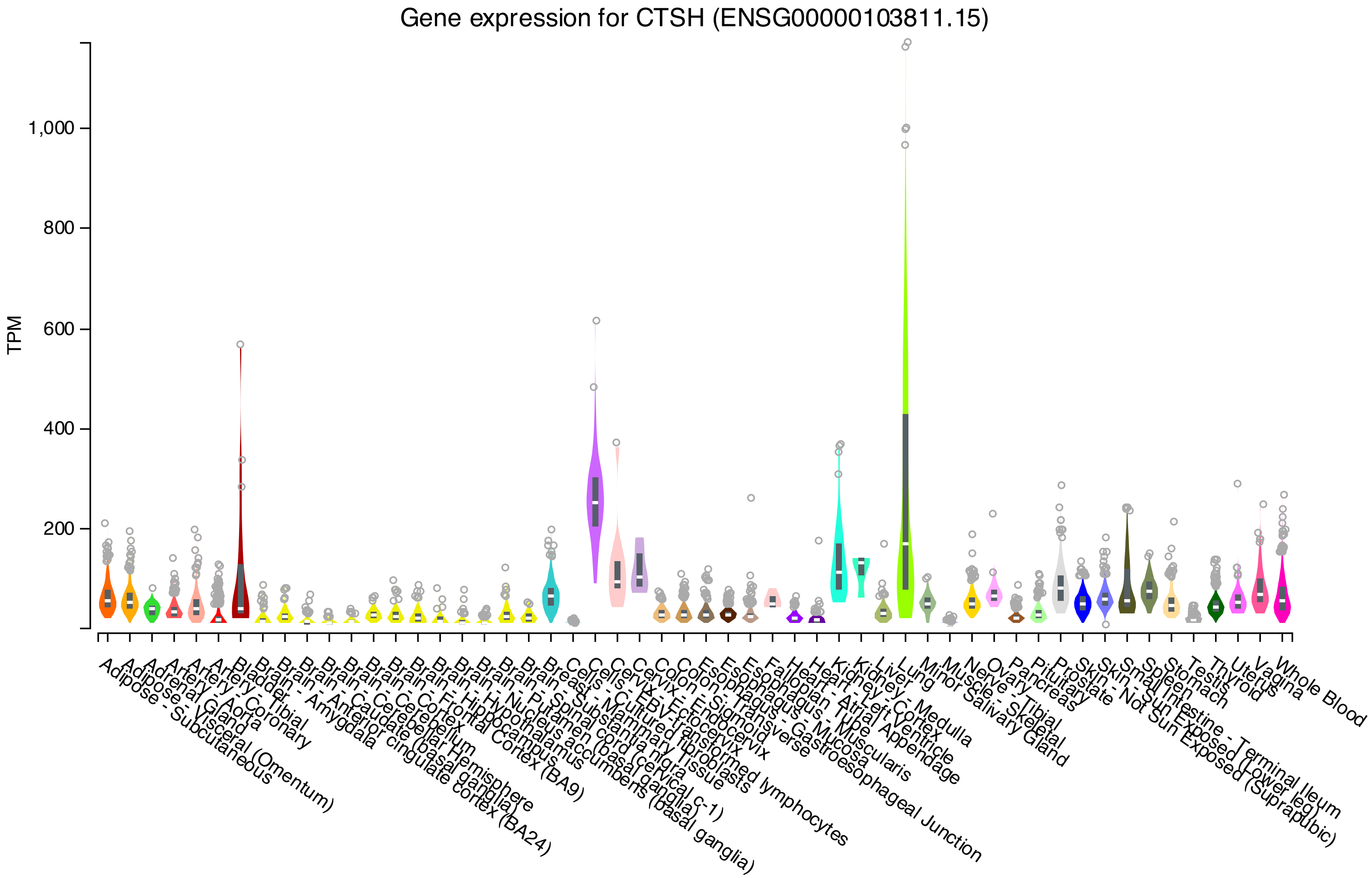

Supplementary Figure 4 – Gene expression profile for *DCC* across 54 tissue types in the GTEx project

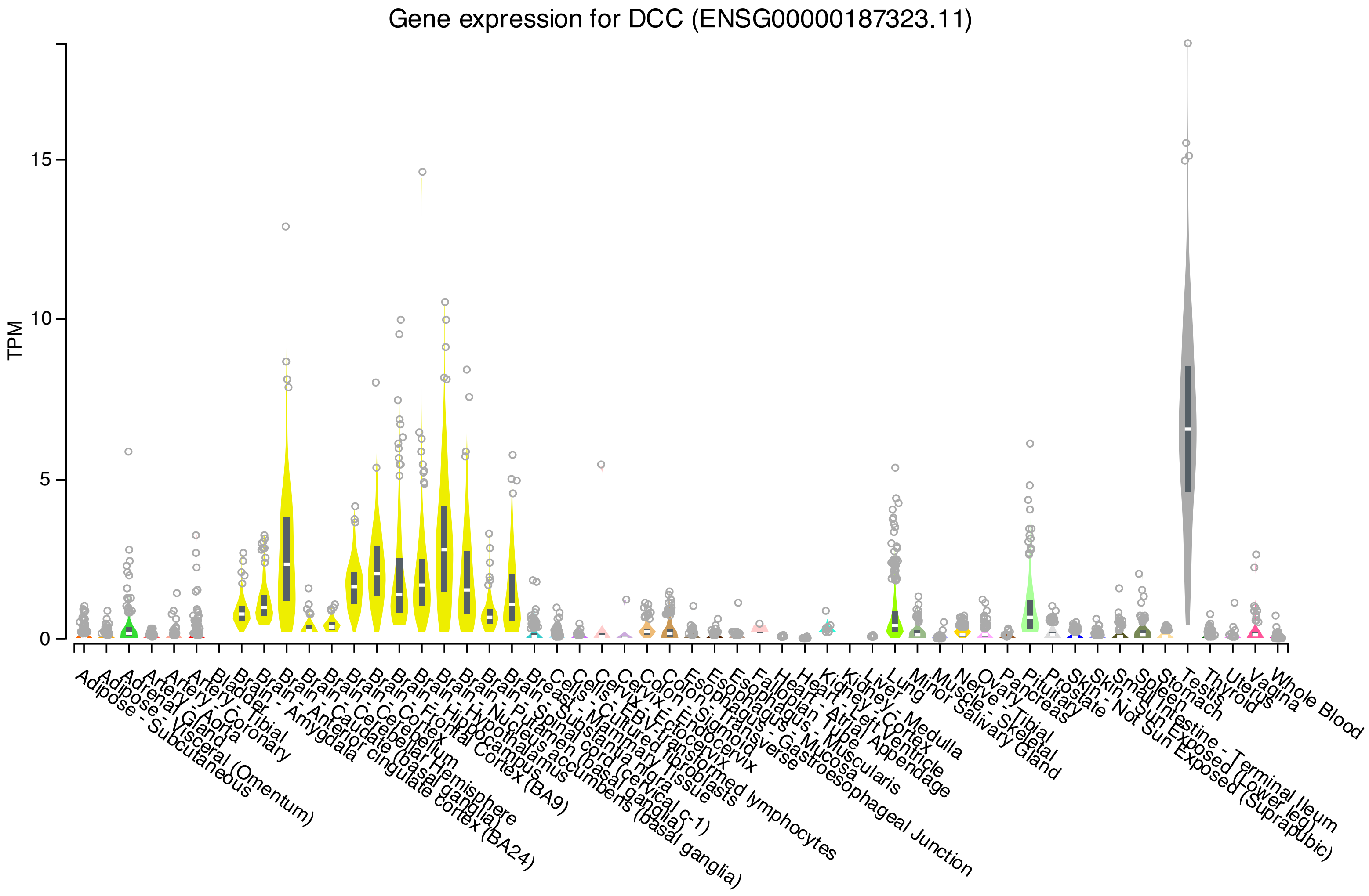

Supplementary Figure 5– Gene expression profile for *ERLIN1* across 54 tissue types in the GTEx project

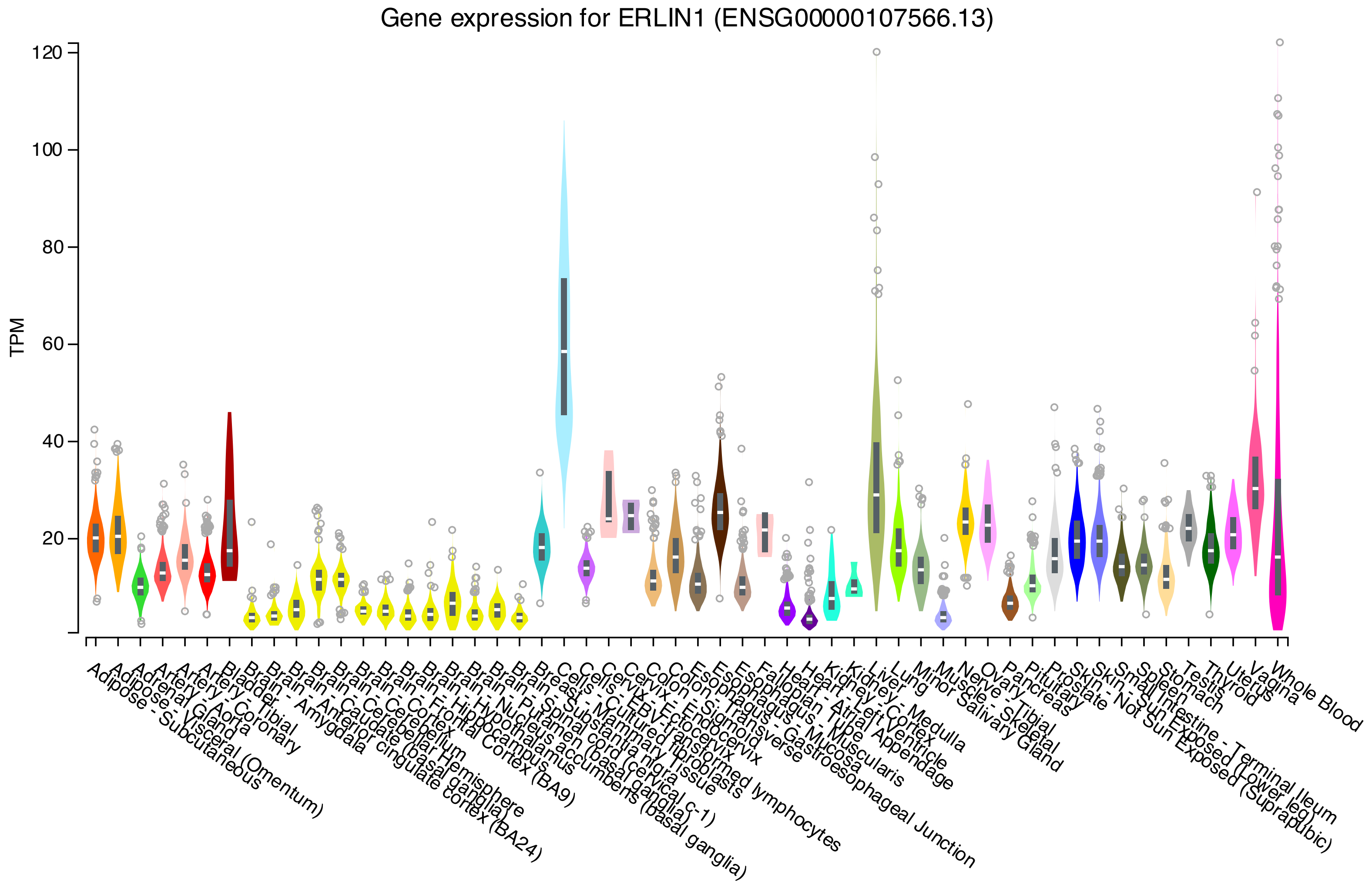

Supplementary Figure 6 – Gene expression profile for *FLOT2* across 54 tissue types in the GTEx project

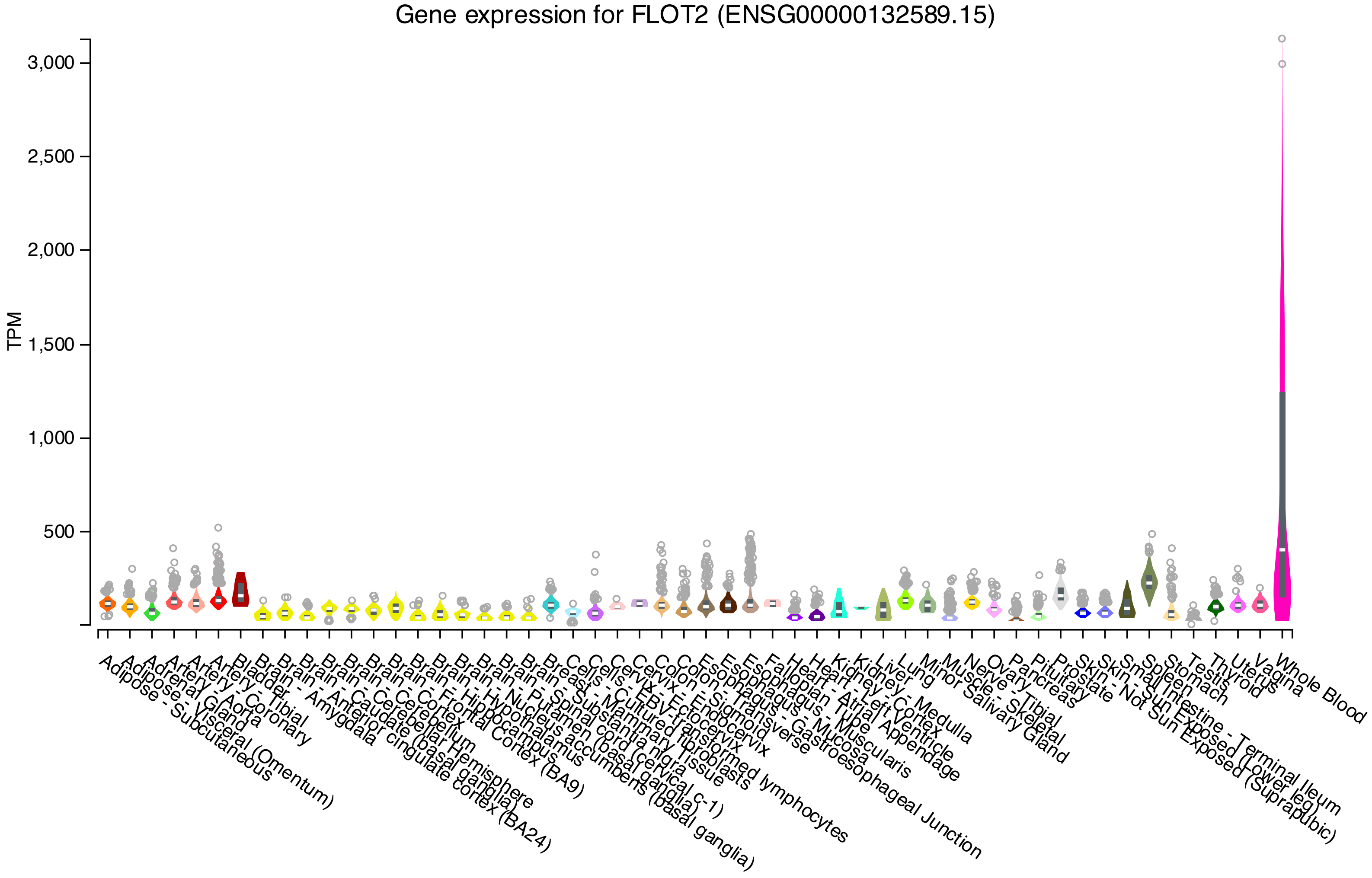

Supplementary Figure 7 – Gene expression profile for *KHK* across 54 tissue types in the GTEx project

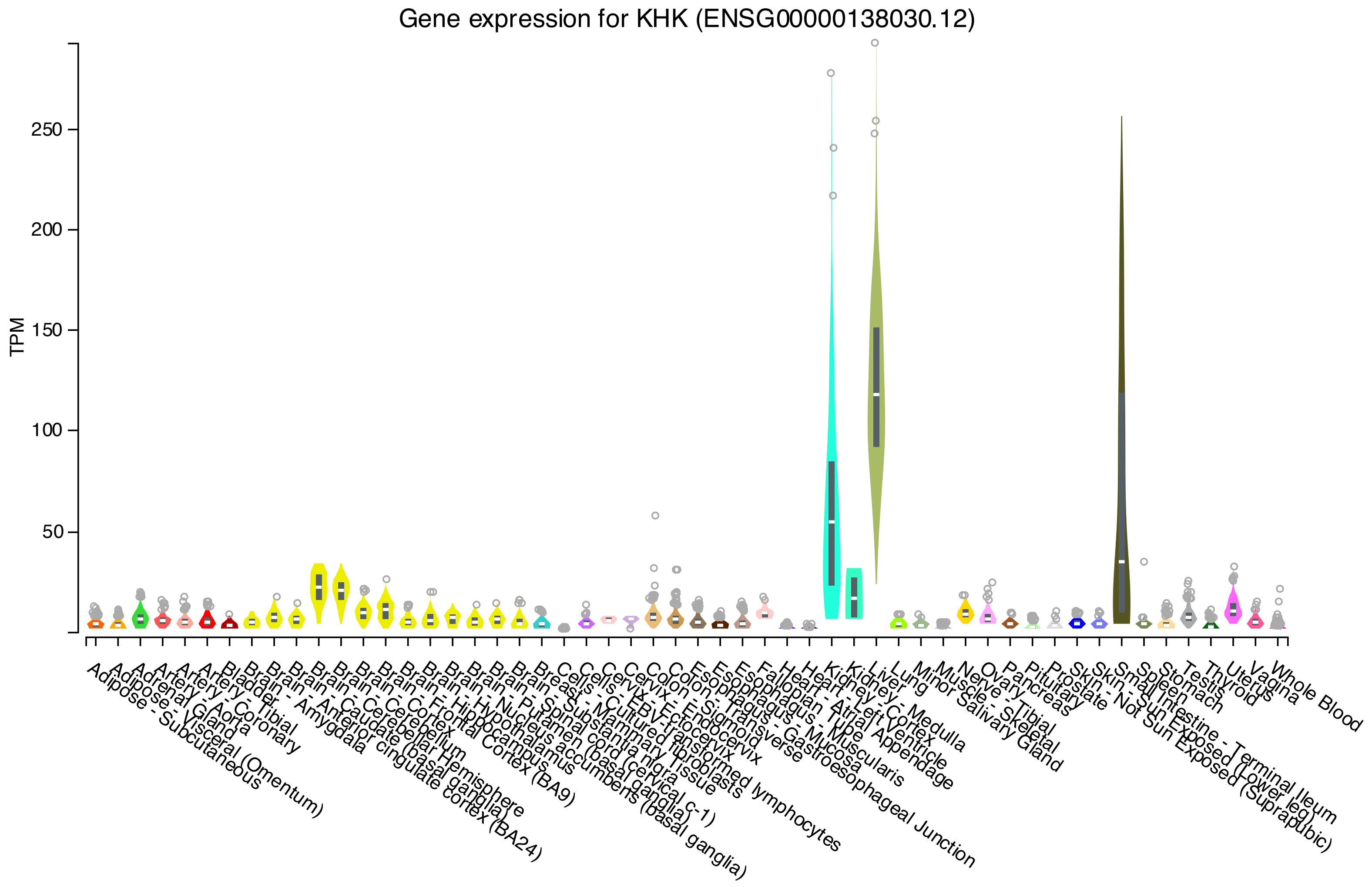

Supplementary Figure 8 – Gene expression profile for *PANK4* across 54 tissue types in the GTEx project

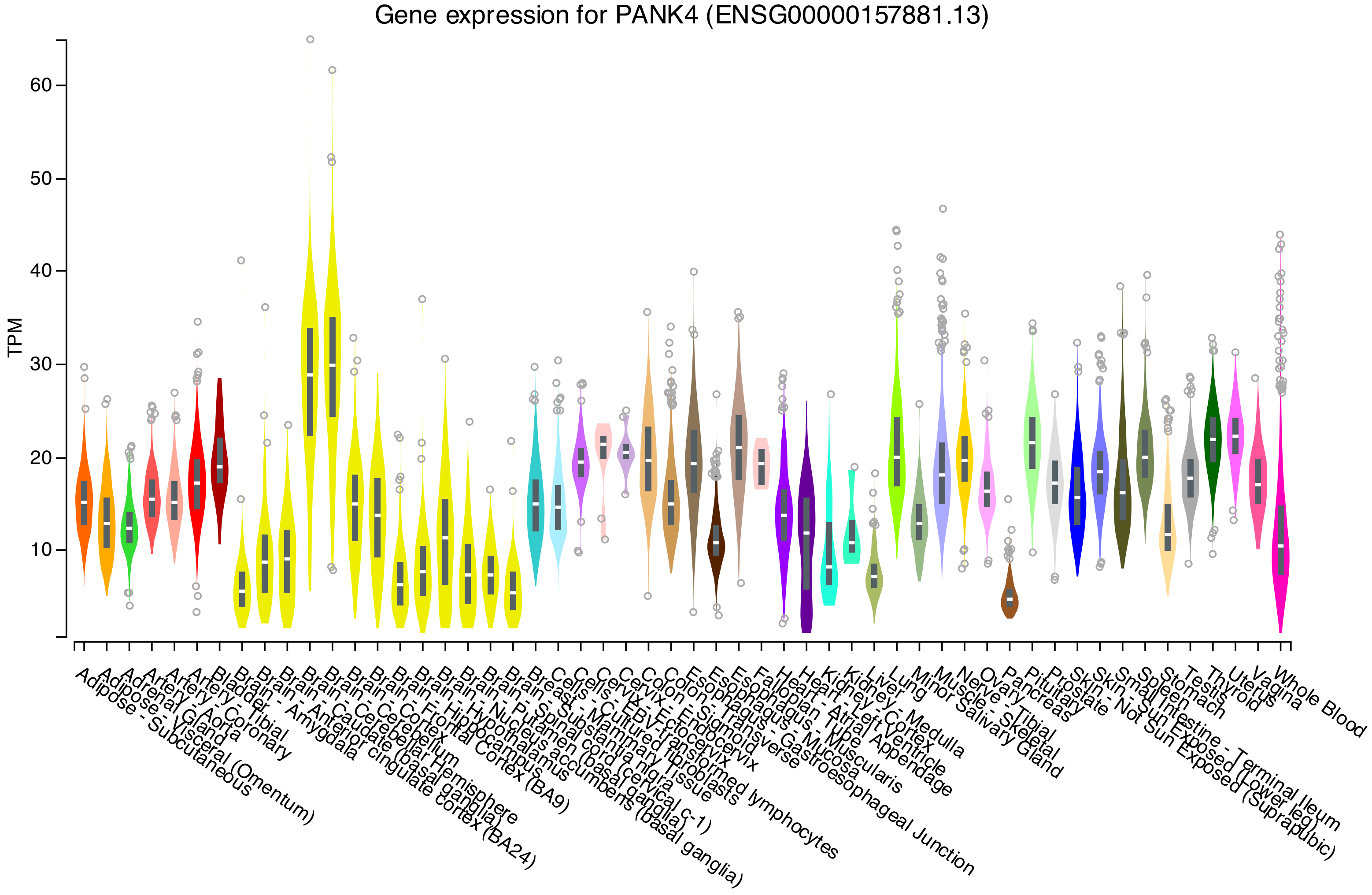

Supplementary Figure 9 – Gene expression profile for *PSMB4* across 54 tissue types in the GTEx project

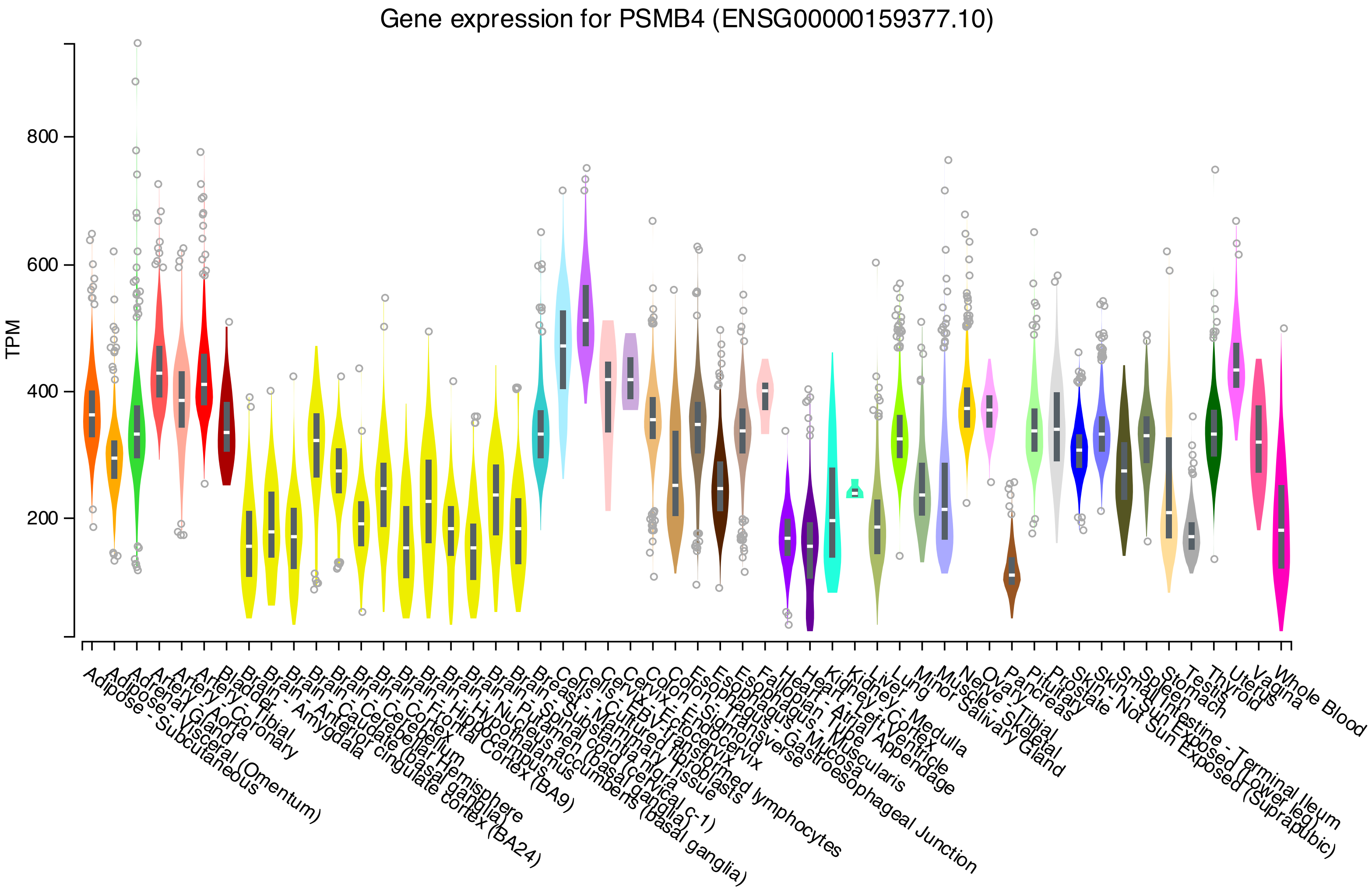

Supplementary Figure 10 – Gene expression profile for *RLBP1* across 54 tissue types in the GTEx project

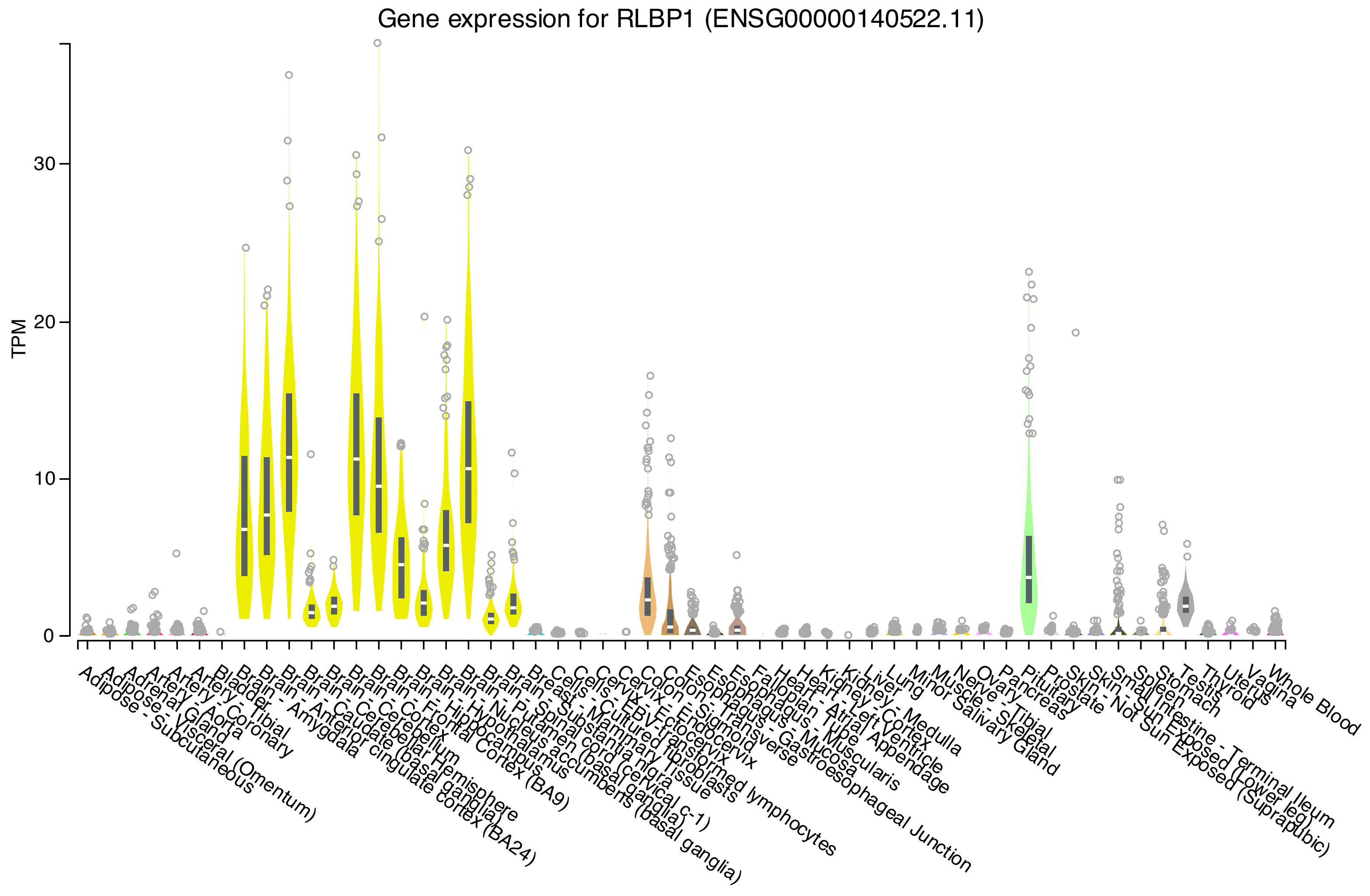

Supplementary Figure 11 – Gene expression profile for *SARM1* across 54 tissue types in the GTEx project

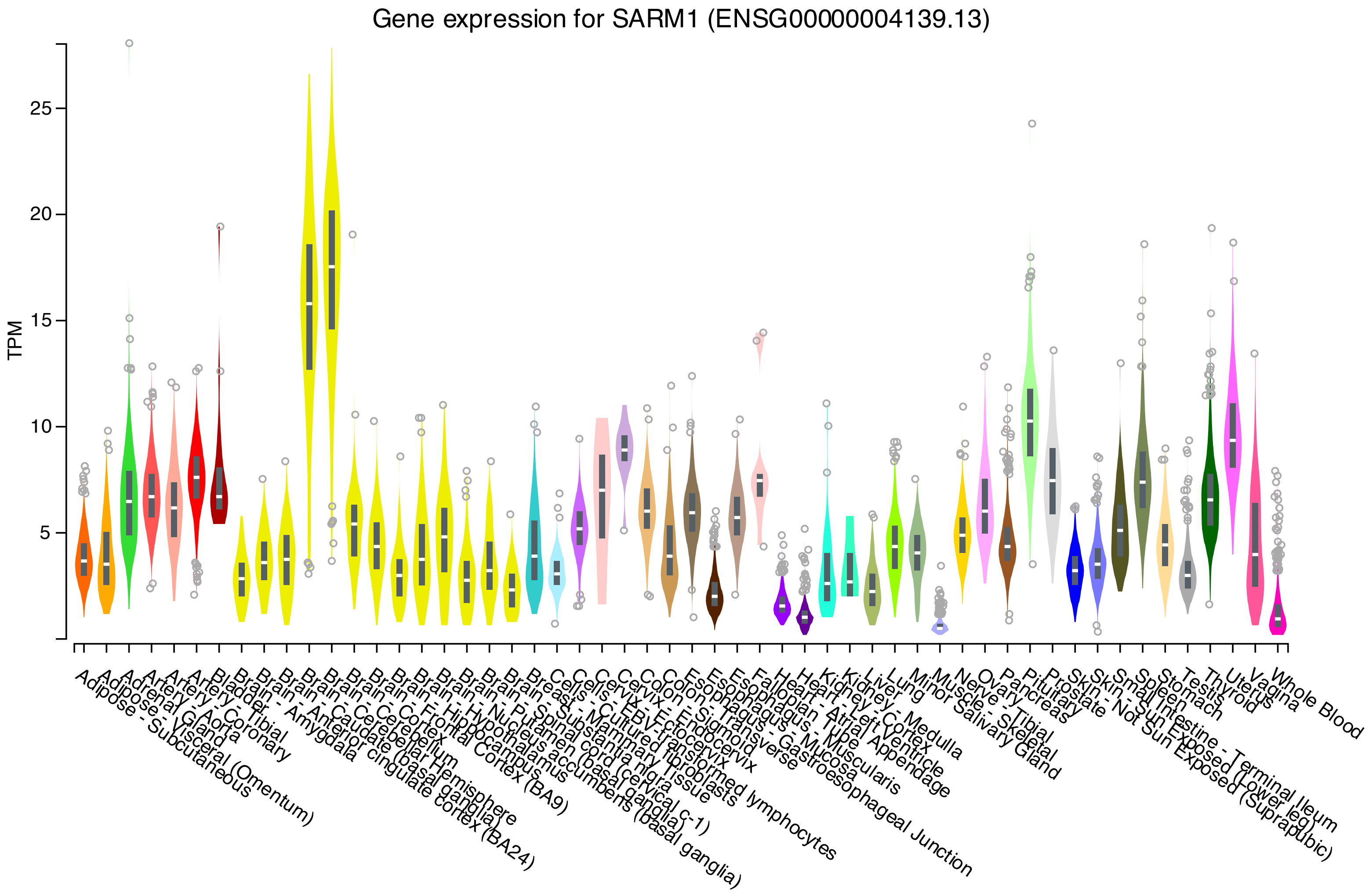

Supplementary Figure 12 – Gene expression profile for *SIDT1* across 54 tissue types in the GTEx project

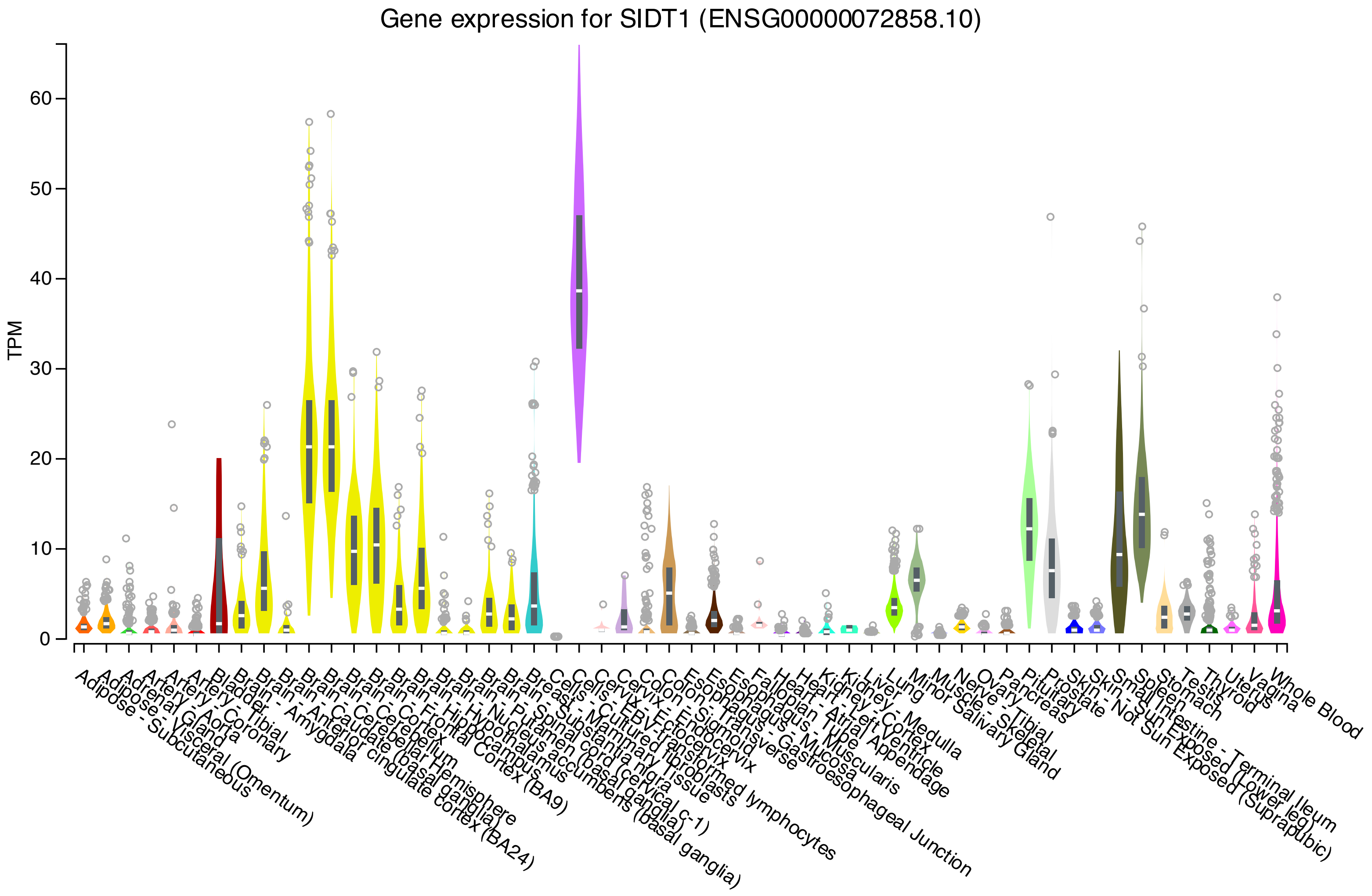

Supplementary Figure 13 – Gene expression profile for *SNX32* across 54 tissue types in the GTEx project

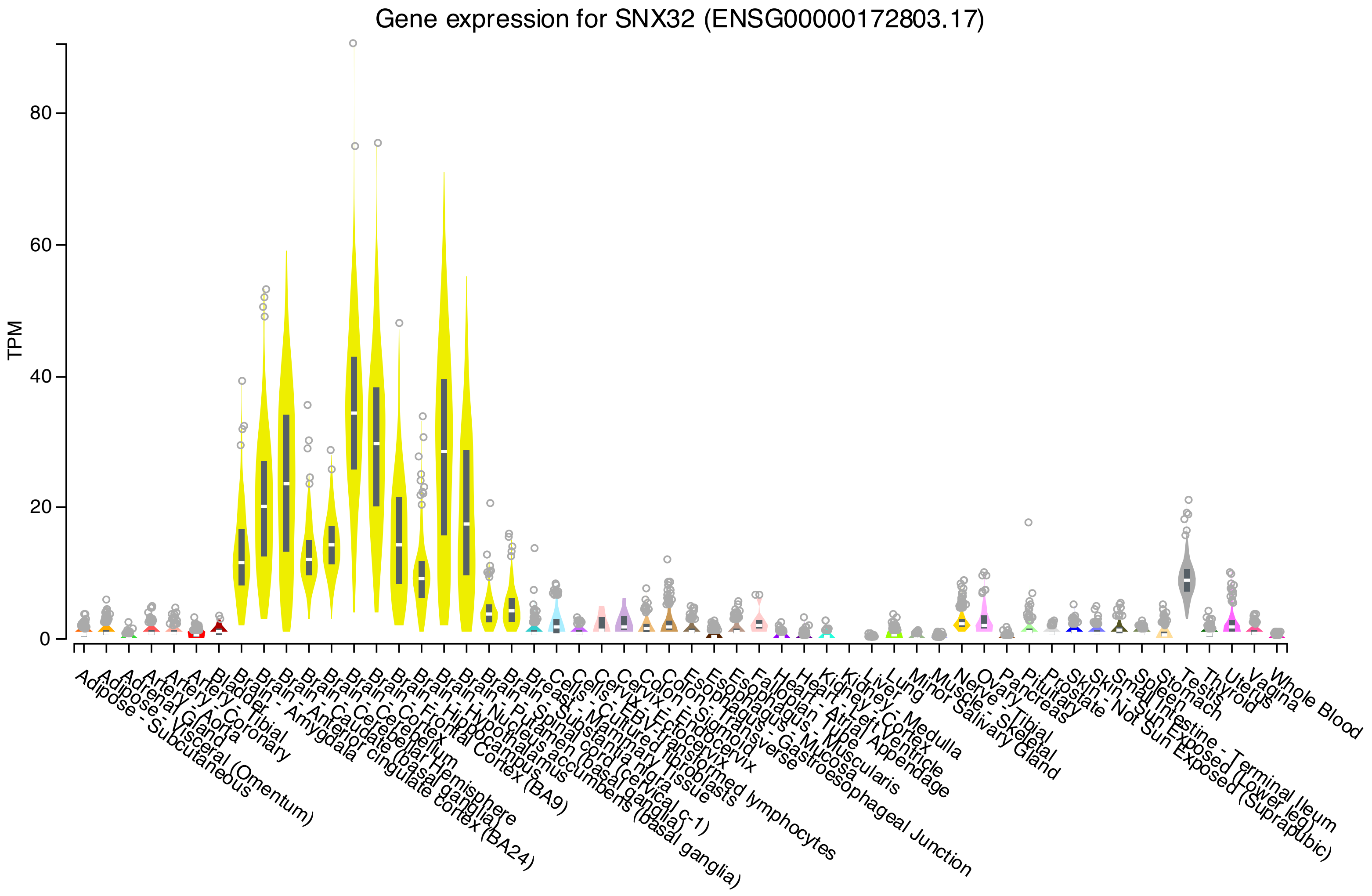

Supplementary Table 1– Characteristics of genome-wide association studies of neurological and psychiatric traits

Trait/disease	Author	Year	URL
Alzheimer's disease	Jansen et al	2019	https://ctg.cncr.nl/software/summary_statistics
Amyotrophic lateral sclerosis	van Rheenen et al	2016	http://databrowser.projectmine.com/
Depression	Howard et al	2019	https://datashare.is.ed.ac.uk/handle/10283/3203
Insomnia	Jansen et al	2018	https://ctg.cncr.nl/software/summary_statistics
Intelligence	Savage et al	2018	https://ctg.cncr.nl/software/summary_statistics
Neuroticism	Elsworth et al [unpublished]	2018	mrbase.org
Schizophrenia	Ruderfer et al	2018	https://www.med.unc.edu/pgc/results-and-downloads
