# Supplementary material for: Characterizing the Causal Pathway for Genetic Variants Associated with Neurological Phenotypes Using Human Brain-Derived Proteome Data
Source: Am J Hum Genet. 2020 May 14;106(6):885–92. doi: 10.1016/j.ajhg.2020.04.007 (PMC7273531; doi:10.1016/j.ajhg.2020.04.007)
Supplement: Document S3 — Article plus Supplemental Information [file mmc3.pdf]

Characterizing the Causal Pathway for Genetic Variants Associated with Neurological Phenotypes Using Human Brain-Derived Proteome Data

Nelson K. Kibinge,[1] Caroline L. Relton,[1] Tom R. Gaunt,[1] and Tom G. Richardson[1,*]

Leveraging high-dimensional molecular datasets can help us develop mechanistic insight into associations between genetic variants and complex traits. In this study, we integrated human proteome data derived from brain tissue to evaluate whether targeted proteins putatively mediate the effects of genetic variants on seven neurological phenotypes (Alzheimer disease, amyotrophic lateral sclerosis, depression, insomnia, intelligence, neuroticism, and schizophrenia). Applying the principles of Mendelian randomization (MR) systematically across the genome highlighted 43 effects between genetically predicted proteins derived from the dorsolateral prefrontal cortex and these outcomes. Furthermore, genetic colocalization provided evidence that the same causal variant at 12 of these loci was responsible for variation in both protein and neurological phenotype. This included genes such as *DCC*, which encodes the netrin-1 receptor and has an important role in the development of the nervous system ($p = 4.29 \times 10^{-11}$ with neuroticism), as well as *SARM1,* which has been previously implicated in axonal degeneration ($p = 1.76 \times 10^{-08}$ with amyotrophic lateral sclerosis). We additionally conducted a phenome-wide MR study for each of these 12 genes to assess potential pleiotropic effects on 700 complex traits and diseases. Our findings suggest that genes such as *SNX32*, which was initially associated with increased risk of Alzheimer disease, may potentially influence other complex traits in the opposite direction. In contrast, genes such as *CTSH* (which was also associated with Alzheimer disease) and *SARM1* may make worthwhile therapeutic targets because they did not have genetically predicted effects on any of the other phenotypes after correcting for multiple testing.

Introduction

The widespread application of genome-wide association studies (GWAS) over the last decade has drastically advanced the discovery of genetic variants associated with complex traits and disease.[1,2] However, the underlying biological mechanisms responsible for the vast majority of these effects have been challenging to decipher.[3] Alterations to circulating protein levels are likely to reside along the causal pathway between genetic variant and phenotypic variation.[4,5] This has led to recent studies characterizing genetic variants associated with protein levels (known as protein quantitative trait loci [pQTL]) by integrating their effects on traits with results from GWAS.[6–10] Furthermore, findings from these endeavors can be valuable for drug target prioritization, particularly given that therapeutic targets with support from genetic association studies are more likely to succeed in clinical trials.[11,12] These estimates have recently been revised, suggesting that support from human genetics can increase approval rates for drugs targeting GWAS traits by over 2-fold.[13]

Circulating protein levels, as with other molecular traits such as gene expression and epigenetic processes, are known to vary depending on the tissue type they are investigated in.[14–16] This has therefore been a limitation for previous studies using human pQTL data, which have typically been confined to plasma proteins derived from whole blood.[6,7] Although these data are more readily accessible in larger samples, they may not necessarily capture biological effects from tissue types that are more relevant to the disease being studied. For example, it would be expected that data derived from brain tissue would be the most pertinent for characterizing genetic variants associated with neurological phenotypes.[17] This is because, due to their functionality in the brain, the underlying genes responsible for these effects are likely to exert their influence on cognitive traits and psychiatric disorders.[18,19]

In this study, we have leveraged pQTL data derived from human brain tissue through the use of the brainQTL resource[20] and findings from large-scale GWAS of seven neurological phenotypes (Alzheimer disease, amyotrophic lateral sclerosis, depression, insomnia, intelligence, neuroticism, and schizophrenia) (Table S1).[21–27] These effects were integrated using a Mendelian randomization (MR) framework which harnesses genetic colocalization to highlight loci where dorsolateral prefrontal cortex proteins and neurological phenotypes are influenced by a shared causal variant. We conducted in-depth evaluations of proteins identified in this analysis by undertaking a phenome-wide MR study to assess their association with 700 complex traits and diseases.

Material and Methods

Data Resources

All genetic effects onto brain-derived protein levels were downloaded from the brainQTL resource (see Web Resources). Further details can be found in the brainQTL paper.[20] In brief, genotype

[1]Medical Research Council (MRC) Integrative Epidemiology Unit (IEU), Population Health Sciences, Bristol Medical School, University of Bristol, Oakfield House, Oakfield Grove, Bristol, BS8 2BN, United Kingdom
*Correspondence: Tom.G.Richardson@bristol.ac.uk
https://doi.org/10.1016/j.ajhg.2020.04.007.
© 2020 The Authors. This is an open access article under the CC BY license (http://creativecommons.org/licenses/by/4.0/).

and proteome data on 7,901 total proteins were available from 144 post-mortem samples from the Religious Orders Study (ROS) and the Memory and Aging Project (MAP).[28] Access to full datasets from the ROS and MAP studies can be requested at the URL in the Web Resources section. pQTL identification was restricted to variants with a minor allele frequency of over 5% and which resided within 100kbs of protein coding genes based on the UCSC genome browser (build GRCh37/hg19).[29]

We obtained genome-wide summary statistics for seven phenotypes by using findings from GWAS for which summary statistics have been made available: Alzheimer disease,[21] amyotrophic lateral sclerosis,[22] depression,[23] insomnia,[24] intelligence,[25] neuroticism,[26] and schizophrenia.[27] More detailed information on all GWAS datasets can be found in Table S1.

Statistical Analysis

Linkage disequilibrium (LD) clumping was undertaken to identify independent pQTL for downstream analyses. This was achieved using PLINK[30] based on a $r^2 < 0.01$ with a reference panel consisting of 10,000 unrelated individuals from the UK Biobank study who were of European descent.[31,32] This reference panel was selected because individuals from the ROS and MAP study were reported to be of European descent.[33] We generated F-statistics for instruments as proposed previously by Bowden et al.:[34]

$$F_j = \frac{\gamma_j^2}{\sigma_{Xj}^2}$$

where γ_j is the SNP-exposure association and σ_{Xj} is the standard deviation for the SNP-exposure association for variant j. Instruments with an F-statistic > 15 were selected to reduce the likelihood of weak instrument bias in downstream analyses.[35]

MR estimates were derived based on the Wald Ratio method[36] using the "TwoSampleMR" package.[37] Estimates were then filtered based on a multiple testing threshold of $p < 0.05$/number of proteins analyzed. Testing of seven neurological phenotypes was not taken into account in this correction, and although some of these share genetic architecture (e.g., schizophrenia and depressive symptoms have previously been reported to have an LD score regression coefficient of $rG = 0.82$[38]), it was important to reinforce results surviving this cut-off with evidence of genetic colocalization. We therefore carried forward the loci that survived our correction threshold from the previous analysis, and we analyzed them with the "coloc" R package[39] and eCAVIAR method[40] using default parameters. Evidence of genetic colocalization was defined as having either a posterior probability of association (PPA) > 0.8 from the coloc method or a colocalization posterior probability (CLPP) > 0.01 for eCAVIAR (both of these were proposed by the developers of these methods). Results that provide evidence based on these parameters suggest that at these loci, brain-derived proteins and neurological phenotypes share a common causal variant.

We subsequently applied this analysis pipeline to 700 complex traits and diseases (a full list of which are in Table S5) by using the TwoSample MR package for any predicted effects which also had evidence of genetic colocalization from the previous analysis. These phenotypes were selected based on the following criteria: (A) outcomes which provided evidence of heritability (based on a heuristic $P_{heritability} < 0.05$) according to analyses undertaken previously by the Neale Lab (see Web Resources) or (B) outcomes from GWAS consortia based on previously defined criteria.[41] Specifically, this included outcomes analyzed by GWAS studies that reported betas, standard error, and effect alleles for over 100,000 genetic variants and that were undertaken in a European population of over 1,000 individuals.

We also performed additional analyses for *SARM1* and *CTSH*, for which our initial analysis did not provide evidence of any putative side effects based on Bonferroni corrections. This was undertaken by comparing the estimates from the analysis of these targets on the 700 outcomes with 500 randomly selected pQTL from our initial sample. We then ranked MR effect estimates and calculated permutated p values based on the rank of *SARM1* and *CTSH* effects compared to those of the other 500 proteins. Any effects which provided evidence based on $P_{permuted} < 0.05$ were further subject to the same genetic colocalization analysis as previously undertaken. Lastly, we investigated evidence of genetic colocalization between pQTL and expression quantitative trait loci (eQTL), also derived from brain tissue, based on analyses by Qi et al. ($n = 1{,}194$).[17]

Results

A Proteome-wide Mendelian Randomization Study of Neurological Traits and Psychiatric Disorders Using Brain-Derived Data

Applying our selection critieria to identify independent pQTL resulted in 692 proteins which were eligible for analysis (Table S2). However, all of these proteins could only be instrumented using a single pQTL, and therefore all MR estimates derived in this study are based on the Wald ratio method.[35] As sample sizes of protein data derived from disease-relevant tissues increase in the future, the MR framework proposed here has the capacity to harness multiple genetic instruments in order to investigate genetically predicted effects.

We systematically applied the principles of MR to generate effect estimates for each of the 692 proteins on each of the seven neurological phenotypes in turn. This identified 43 genetically predicted effects based on a multiple testing threshold of $p < 7.23\text{x}10^{-05}$ (i.e., 0.05/692 proteins) (Table S3). To support evidence of an effect between proteins and outcomes, we applied genetic colocalization methods to discern whether the causal pQTL at these loci was also responsible for variation in neurological phenotypes.

This identified 12 loci which provided evidence of genetic colocalization based on a PPA > 0.8 for the coloc method or a CLPP > 0.01 for eCAVIAR results (Table S4). Each of these effects has been highlighted on the Manhattan plot in Figure 1. A flowchart illustrating the overall analysis pipeline applied in this study can be found in Figure S1.

Genetic Colocalization Helps Develop Insight at Genome-wide Association Loci and Highlights Potentially Novel Signals

Amongst the results with evidence of genetic colocalization were GWAS loci which harbor genes thought to be involved in neurological functionality. For example, the *DCC* locus, which was associated with neuroticism

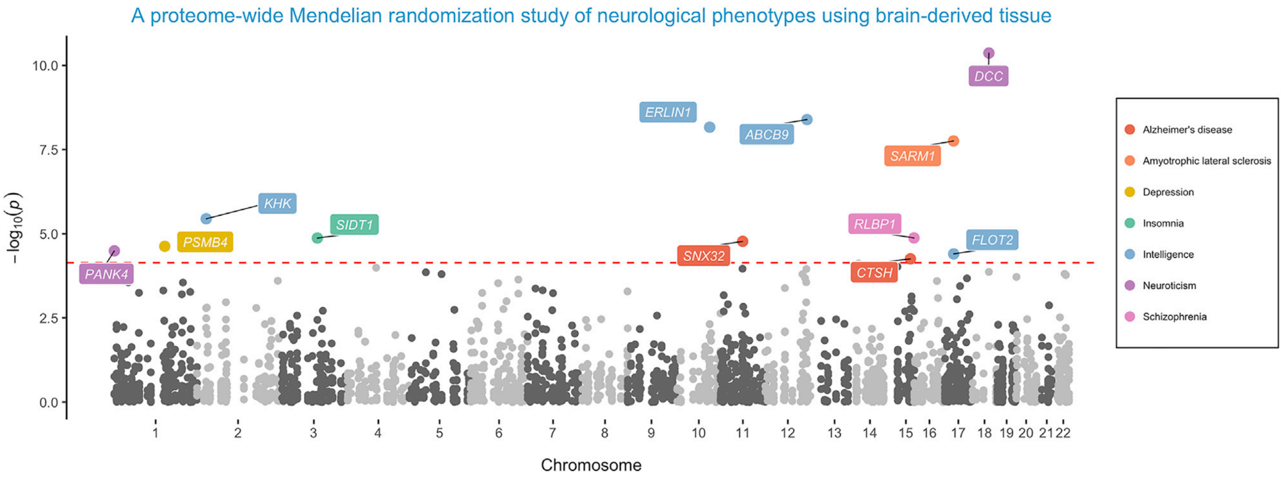

Figure 1. A Manhattan Plot to Highlight Genetically Predicted Effects Based on Mendelian Randomization and Genetic Colocalization Analyses on Neurological Phenotypes

Points correspond to the −log10 p values that reflect genetically predicted effects between protein quantitative trait loci and neurological phenotypes. The red dashed line indicates the multiple testing correction applied in analyses ($p = 0.05/692 = 7.23 \times 10^{-05}$). Effects that surpassed this threshold were only included in this plot if they also provided evidence of genetic colocalization, and these effects are colored based on their associated traits.

risk in our analysis ($p = 4.29 \times 10^{-11}$), encodes the netrin-1 receptor, which has been reported to play a role in the development of the nervous system. Similarly, *SARM1* was associated with amyotrophic lateral sclerosis risk ($p = 1.76 \times 10^{-08}$) and has been previously reported to influence axonal degeneration.[42] Integrating brain-derived protein data at these GWAS loci can therefore provide insight into the biological pathway by which the underlying causal variants influence these neurological phenotypes.

In contrast, other loci highlighted by our findings are putatively novel trait-associated variants that have not yet reached genome-wide evidence thresholds (i.e., $p < 5 \times 10^{-08}$). As such, evidence of genetic colocalization with pQTL derived from a tissue type relevant to these phenotypes can be valuable in terms of prioritizing loci that are yet to be uncovered by GWAS. Two examples of this include *FLOT2* ($p = 3.97 \times 10^{-05}$ with intelligence), which encodes the neuronal signaling factor flotillin-2, and *SIDT1* ($p = 1.34 \times 10^{-05}$ with insomnia), which is a dsRNA transporter. Figure 2 illustrates evidence of colocalization at both of these loci with their respective neurological phenotypes and dorsolateral prefrontal cortex proteins. Whereas these signals are likely to be uncovered by GWAS once sample sizes increase, evidence that they colocalize with disease-relevant protein data may shed light on the causative pathway responsible for these signals. We postulate that future endeavors adopting a similar approach but using larger omic datasets will possess increased power to elucidate putatively novel findings.

Prioritizing Therapeutic Targets by Undertaking a Phenome-wide Mendelian Randomization Study

For each of the 12 loci highlighted by our initial analysis, we conducted a phenome-wide MR analysis to evaluate putative pleiotropic effects on 700 complex traits and diseases (Table S5). Based on multiple testing comparisons (i.e., $p < 0.05/700 = 7.14 \times 10^{-05}$), along with the same genetic colocalization thresholds used previously, there was evidence of pleiotropy at various loci. For example, based on these criteria, the lead pQTL at *SNX32* colocalized with 12 different phenotypes along with Alzheimer disease in the initial analysis (with $p = 1.68 \times 10^{-05}$). As depicted in Figure 3A, therapeutically targeting this gene to reduce risk of Alzheimer disease is genetically predicted to influence other outcomes in the opposite direction, such as HDL cholesterol levels ($p = 3.14 \times 10^{-05}$) and body fat percentage ($p = 2.51 \times 10^{-05}$). Further evaluations of this target are necessary to discern whether increased genetic liability toward Alzheimer disease risk is responsible for these predicted effects (e.g., lower adiposity). Alternatively, genetic variation at this locus may influence these outcomes separately via alternate biological pathways (also known as "horizontal pleiotropy"), which may make *SNX32* less attractive as a therapeutic target.

In contrast, there were several genes which did not provide evidence of pleiotropy based on this analysis. For instance, the lead pQTL for *SARM1*, which had an effect on amyotrophic lateral sclerosis risk in the initial analysis ($p = 1.76 \times 10^{-08}$), did not provide evidence of an effect with any of the 700 outcomes assessed based on multiple testing corrections (Figure 3B). We further explored evidence of potential side effects for *SARM1* by comparing the distribution of its MR estimates on all 700 traits with those of 500 randomly selected pQTL. The strongest evidence for a secondary effect potentially overlooked by Bonferroni corrections was on coronary artery disease ($P_{permutation} = 0.004$). However, this effect was not

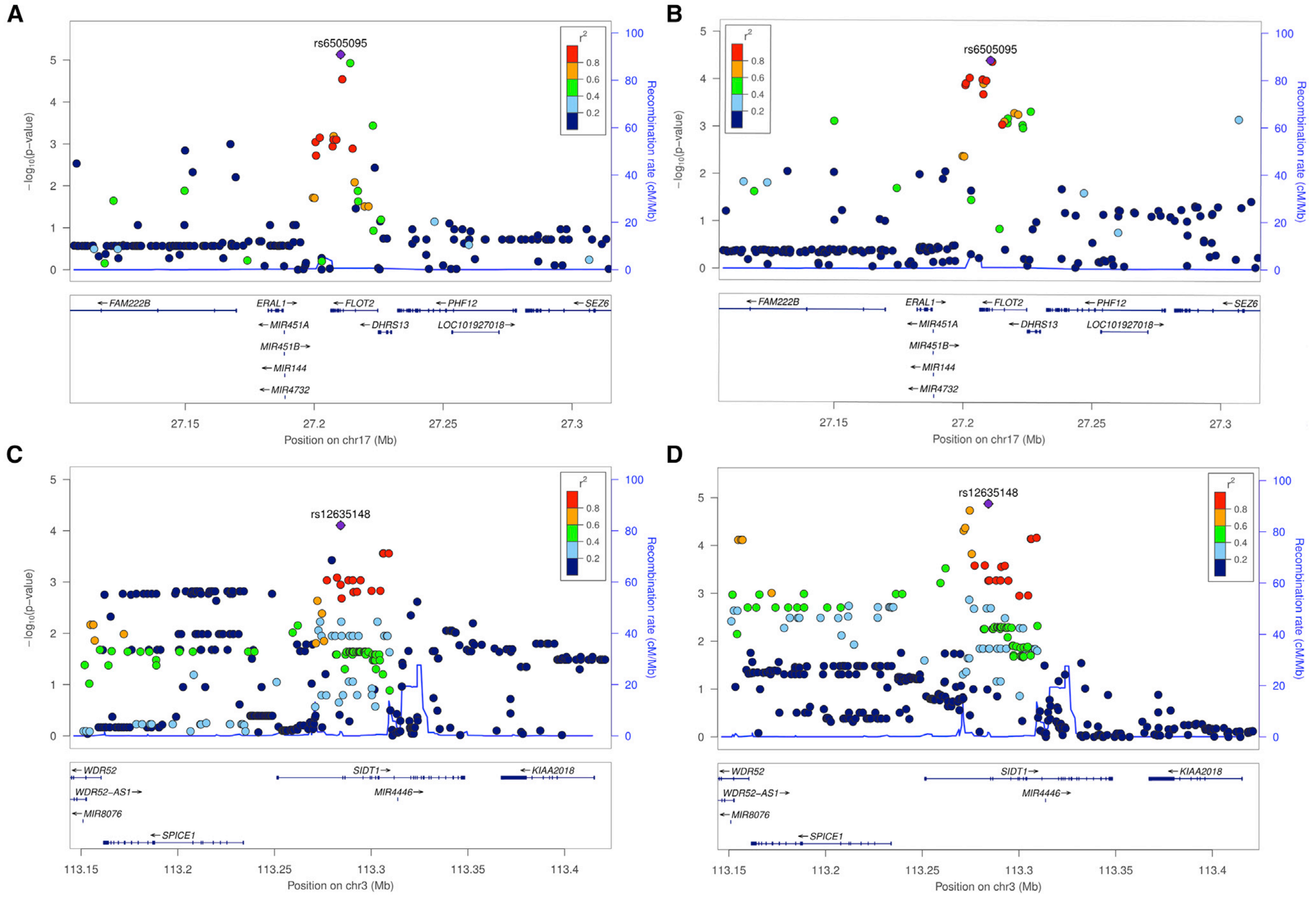

Figure 2. Locuszoom Plots to Illustrate Evidence of Genetic Colocalization between Proteins and Neurological Phenotypes
Regional −log10 p values at the *FLOT2* locus on (A) flottilin-2 levels and (B) intelligence and also at the *SIDT1* locus on (C) SIDT1 protein levels and (D) insomnia.

supported by evidence of colocalization (PPA = 12.7% and CLPP = 2.92×10^{-04}), and this effect had an FDR of 0.06.

Additionally, the only effect surviving Bonferroni corrections for *CTSH*, which encodes the cathepsin H protein, were on standing and sitting height ($p = 4.28 \times 10^{-05}$ and $p = 1.49 \times 10^{-05}$ respectively). However, these effects were not supported by evidence of genetic colocalization. Examples such as *SARM1* and *CTSH* should therefore be prioritized as worthwhile candidates for therapeutic intervention, given that a lack of pleiotropic effects from this analysis using human genetics supports their safety and efficacy.

Discussion

We have conducted a study to characterize genetic variants associated with neurological phenotypes by harnessing brain-derived protein data. Under the principles of MR, we identified 43 genetically predicted effects across the genome, and this suggested that there may be a shared genetic architecture between neurological phenotypes and the subset of proteins studied. Applying two different genetic colocalization techniques provided evidence that effects at 12 of these loci were driven by a common causal variant. We next undertook a phenome-wide association study for each of these 12 proteins by applying this approach systematically to 700 complex traits and disease endpoints. Doing so elucidated pleiotropic proteins associated with various outcomes, along with protein targets associated more specifically with their corresponding neurological phenotype as identified in our initial analysis.

The influx of high-dimensional datasets concerning intermediate phenotypes provides an exceptional opportunity to unravel the biological mechanisms responsible for GWAS signals.[43] The tissue type used to capture these molecular signatures has been shown to play an important role in such endeavors.[44] For instance, previous comparisons of quantitative trait loci associated with the same gene target identified a correlation of $r^2 = 0.70$ between brain and whole blood.[17] While this suggests that blood may act as a valid proxy for brain tissue the majority of the time, there may be effects that would potentially be overlooked by not using the most pertinent tissue type for the investigated GWAS trait. As an example of this, the fine-mapped pQTL for *ERLIN1* in this study shows no evidence on an effect on this protein in whole blood based on the most comprehensive plasma protein QTL analysis to

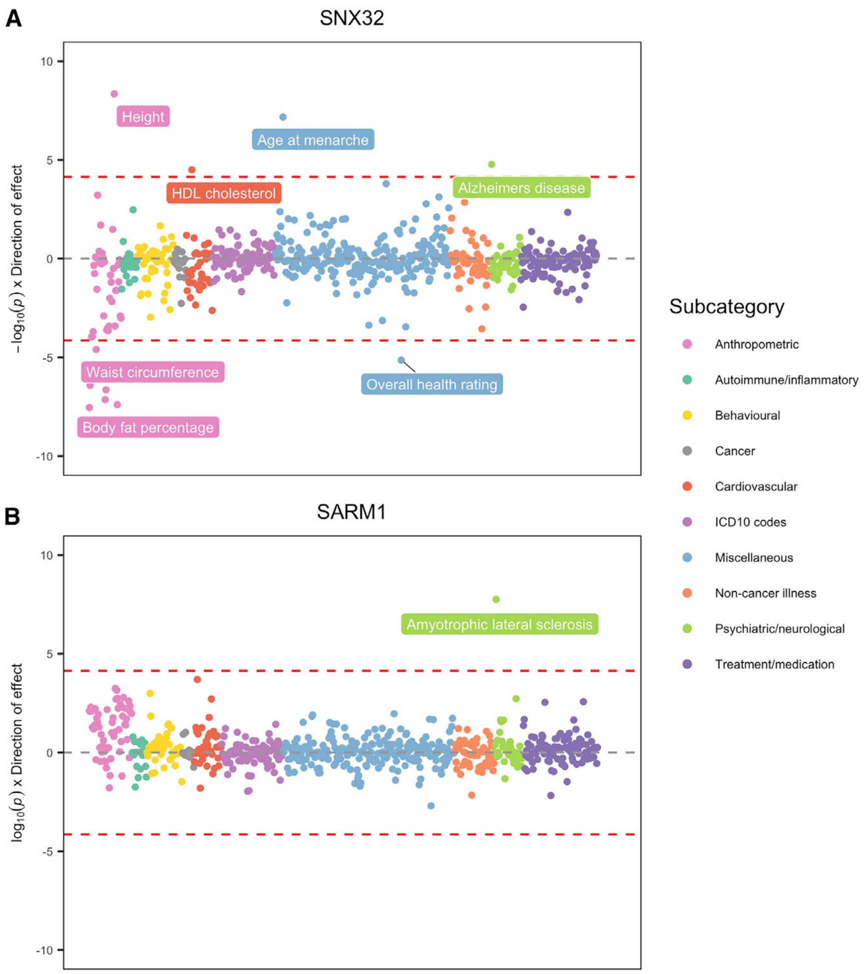

Figure 3. Phenome-wide Association Plots for (A) SNX32 and (B) SARM1 to Investigate Pleiotropic Effects
Each point on these plots corresponds to the –log10 p values derived using the Wald ratio, which are clustered and colored based on the subcategory of each trait and oriented to reflect the direction of effect with each respective protein. Red dashed lines correspond to the multiple testing correction threshold of $p < 0.5/700 = 7.14 \times 10^{-05}$.

date by Sun et al.[6] (rs11190393, $p = 0.92$). In fact, the only fine-mapped pQTL we were able to replicate using this data was for *CTSH* (rs34593439), although this protein has yet to be linked with Alzheimer disease in whole blood analyses based on evidence from the EpiGraphdb platform (URL located in the Web Resources section).

Among the loci highlighted in our study are various genes which have been previously reported to play a role in brain-related activities. This includes *DCC*, which is associated with neuroticism in our analyses ($p = 4.29 \times 10^{-11}$) and is responsible for expression of the nectin-1 receptor. Nectin-1 has previously been implicated in various neurological and psychiatric disorders, including schizophrenia and depression.[45] Similarly, *FLOT2*, which was associated with intelligence ($p = 3.97 \times 10^{-05}$), encodes neuronal signaling factor flotillin-2, and it has been linked previously with autism and related disorders.[46] Elsewhere, *PSMB4*, which encodes a member of the proteasome B-type family, was associated with depression risk ($p = 2.36 \times 10^{-05}$). Proteasomes have been implicated previously in risk of neurodegenerative disorders.[47,48] These findings may therefore help shed some light on the causal pathway between trait-associated genetic variants at these loci and their respective phenotypes.

Characterizing GWAS signals using tissue-relevant data can also be valuable for translational purposes such as prioritizing therapeutic targets. In particular, *SARM1* ($p = 1.76 \times 10^{-08}$ with amyotrophic lateral sclerosis) and *CTSH* ($p = 5.57 \times 10^{-05}$ with Alzheimer disease) represent the most promising candidates based on our evaluations. This is because our phenome-wide analyses did not detect strong evidence of pleiotropic effects on non-neurological traits, which may foreshadow adverse side-effects from targeting these genes or their mechanism of action using therapeutics. Evidence from the literature has reported that genetic deletion of *SARM1* in mice can block pathological axon degeneration.[49,50] Therapeutically inhibiting *SARM1* may therefore be a putatively viable strategy for treating neurodegenerative diseases characterized by axon loss, such as amyotrophic lateral sclerosis.[42] *CTSH* has also previously been linked with Alzheimer disease, where its expression in the temporal cortices of late-onset Alzheimer patients was shown to be altered.[51] Although our phenome-wide association study included outcomes which are not clinically relevant (e.g., height), the purpose of this “hypothesis-free” analysis was to prioritize potential targets based on overall pleiotropic effects. For example, a target predicted to influence non-clinically relevant endpoints may still be more attractive than one linked only to the target disease being evaluated. It is likely that the majority of therapeutic targets will result in some type of unanticipated side effect, which means that anyone assessing this based on human genetics should primarily be concerned with evaluating whether predicted adverse effects outweigh any potential benefit.

We found, based on evaluations in the GTEx project across 54 tissue types,[52] that various loci highlighted in our analyses were predominantly expressed in brain tissue (Figures S2–S13). However, in our extended analyses based on a previously meta-analyzed sample of 1,194 individuals, only four of the 12 identified proteins provided

evidence of genetic colocalization with gene expression (*CTSH*, *KHK*, *PSMB4,* and *SNX32,* Table S7). There could be various reasons for this lack of agreement, which has been reported previously by the authors of the brainQTL resource,[20] such as technical artifacts in assays. There may also be biological explanations such as canalization, the phenomenon used to describe the robustness of phenotypic characteristics in the presence of abundant genetic variation and environmental conditions.[53,54] This lack of evidence for the eight proteins which did not colocalize appeared to be due to the fact that their lead pQTL were not also eQTL in the meta-analyzed dataset. However, based on findings from the eQTLGen consortium ($n = 31,684$), we did find that 10 of the 12 fine-mapped pQTL for these proteins are strongly associated with their corresponding genes' expression in whole blood. The two exceptions were *DCC* and *RLBP1*, which were not analyzed by eQTLGen, and GTEx evaluations suggested that they may not be strongly expressed in whole blood (Figures S4 and S10). As such, a higher proportion of transcriptomic and proteomic signatures may colocalize once sample sizes of brain-derived molecular datasets increase.

This highlights the key limitation of our study, which is the current sample size of accessible proteome-wide data derived from brain tissue ($n = 144$ from the brainQTL resource). This limited the number of proteins we were able to instrument using pQTL and also meant we were confined to using single-pQTL instruments. Furthermore, it reduced the overall statistical power of the initial pQTL study, which had downstream implications for our colocalization analysis in terms of the number of signals which met conventional thresholds. Future endeavors which continue to uncover the genetic architecture of the human proteome in disease-relevant tissue types will improve our capability to reliably instrument them under the principle of MR. This will also improve the robustness of evidence from genetic colocalization analyses.

We also found that the two genetic colocalization techniques used in this study (the coloc and eCAVIAR approaches) did not always provide corroborating evidence. For example, the loci highlighted in Figure 2 were identified only when using the eCAVIAR approach, despite these plots providing graphical illustrations of genetic colocalization to support findings. A possible explanation for this is the sensitivity of prior distributions selected for the coloc method, which therefore supports our decision to apply multiple methods which make different assumptions about the underlying genetic architecture of a region. However, although genetic colocalization can help support evidence of causality by reducing the likelihood that LD has influenced findings, it cannot rule out horizontal pleiotropy. This is the phenomenon whereby two traits (e.g., a circulating protein and neurological phenotype) are influenced by the same causal variant but by via two independent biological pathways. Functional follow-up work with the proteins highlighted in this work is therefore necessary in order to robustly investigate this.

In conclusion, the findings from this study can help elucidate the causal pathway for genetic variants associated with neurological phenotypes and also prioritize candidate targets for therapeutic intervention. Future studies which leverage increasingly large-scale molecular datasets derived from disease-relevant tissues will continue to develop insights into the mechanisms linking genetic variation to complex traits and disease.

Supplemental Data

Supplemental Data can be found online athttps://doi.org/10.1016/j.ajhg.2020.04.007.

Acknowledgments

We would like to thank the developers of the brainqtl.org resource, the participants of the ROS and MAP studies, and the GWAS consortia for making their summary statistics available for the benefit of this study. This work was supported by the Integrative Epidemiology Unit, which receives funding from the UK Medical Research Council and the University of Bristol (MC_UU_00011/4 and MC_UU_00011/5). C.L.R and T.R.G conduct research at the National Institute for Health Research (NIHR) Biomedical Research Centre at the University Hospitals Bristol National Health Service (NHS) Foundation Trust and the University of Bristol. The views expressed in this publication are those of the author(s) and not necessarily those of the NHS, the NIHR, or the Department of Health. T.G.R is a UK Research and Innovation (UKRI) Research Fellow (MR/S003886/1). The reference panel used in the study was created using data from the UK Biobank study (app 15825).

Declaration of Interests

T.R.G. and C.L.R have previously received research funding from Sanofi. T.G.R. and N.K. have both been previously funded by Sanofi. T.R.G receives research funding from GlaxoSmithKline and Biogen. However, none of these factors contributed to the design or analysis of this study.

Received: January 31, 2020
Accepted: April 6, 2020
Published: May 7, 2020

Web Resources

BrainQTL resource,https://brainqtl.org/
EpiGraphdb,www.epigraphdb.org/pqtl
Neale Lab analyses in the Biobank UK,http://www.nealelab.is/uk-biobank
ROS and MAP studies,https://www.radc.rush.edu/

References

1. Visscher, P.M., Wray, N.R., Zhang, Q., Sklar, P., McCarthy, M.I., Brown, M.A., and Yang, J. (2017). 10 Years of GWAS Discovery: Biology, Function, and Translation. Am. J. Hum. Genet. *101*, 5–22.

2. Trenkmann, M. (2018). Lessons from 1 million genomes. Nat. Rev. Genet. *19*, 592–593.
3. Cannon, M.E., and Mohlke, K.L. (2018). Deciphering the Emerging Complexities of Molecular Mechanisms at GWAS Loci. Am. J. Hum. Genet. *103*, 637–653.
4. Gallagher, M.D., and Chen-Plotkin, A.S. (2018). The Post-GWAS Era: From Association to Function. Am. J. Hum. Genet. *102*, 717–730.
5. Schaid, D.J., Chen, W., and Larson, N.B. (2018). From genome-wide associations to candidate causal variants by statistical fine-mapping. Nat. Rev. Genet. *19*, 491–504.
6. Sun, B.B., Maranville, J.C., Peters, J.E., Stacey, D., Staley, J.R., Blackshaw, J., Burgess, S., Jiang, T., Paige, E., Surendran, P., et al. (2018). Genomic atlas of the human plasma proteome. Nature *558*, 73–79.
7. Emilsson, V., Ilkov, M., Lamb, J.R., Finkel, N., Gudmundsson, E.F., Pitts, R., Hoover, H., Gudmundsdottir, V., Horman, S.R., Aspelund, T., et al. (2018). Co-regulatory networks of human serum proteins link genetics to disease. Science *361*, 769–773.
8. Folkersen, L., Fauman, E., Sabater-Lleal, M., Strawbridge, R.J., Frånberg, M., Sennblad, B., Baldassarre, D., Veglia, F., Humphries, S.E., Rauramaa, R., et al.; IMPROVE study group (2017). Mapping of 79 loci for 83 plasma protein biomarkers in cardiovascular disease. PLoS Genet. *13*, e1006706.
9. McGowan, L.M., Davey Smith, G., Gaunt, T.R., and Richardson, T.G. (2019). Integrating Mendelian randomization and multiple-trait colocalization to uncover cell-specific inflammatory drivers of autoimmune and atopic disease. Hum. Mol. Genet. *28*, 3293–3300.
10. Zheng, J., Haberland, V., Baird, D., Walker, V., Haycock, P., Gutteridge, A., Richardson, T.G., Staley, J., Elsworth, B., Burgess, S., et al. (2019). Phenome-wide Mendelian randomization mapping the influence of the plasma proteome on complex diseases. bioRxiv. https://doi.org/10.1101/627398.
11. Nelson, M.R., Tipney, H., Painter, J.L., Shen, J., Nicoletti, P., Shen, Y., Floratos, A., Sham, P.C., Li, M.J., Wang, J., et al. (2015). The support of human genetic evidence for approved drug indications. Nat. Genet. *47*, 856–860.
12. Cully, M. (2015). Target validation: Genetic information adds supporting weight. Nat. Rev. Drug Discov. *14*, 525.
13. King, E.A., Davis, J.W., and Degner, J.F. (2019). Are drug targets with genetic support twice as likely to be approved? Revised estimates of the impact of genetic support for drug mechanisms on the probability of drug approval. PLoS Genet. *15*, e1008489.
14. Thul, P.J., and Lindskog, C. (2018). The human protein atlas: A spatial map of the human proteome. Protein Sci. *27*, 233–244.
15. Zhang, B., Zhou, Y., Lin, N., Lowdon, R.F., Hong, C., Nagarajan, R.P., Cheng, J.B., Li, D., Stevens, M., Lee, H.J., et al. (2013). Functional DNA methylation differences between tissues, cell types, and across individuals discovered using the M&M algorithm. Genome Res. *23*, 1522–1540.
16. Richardson, T.G., Hemani, G., Gaunt, T.R., Relton, C.L., and Davey Smith, G. (2020). A transcriptome-wide Mendelian randomization study to uncover tissue-dependent regulatory mechanisms across the human phenome. Nat. Commun. *11*, 185.
17. Qi, T., Wu, Y., Zeng, J., Zhang, F., Xue, A., Jiang, L., Zhu, Z., Kemper, K., Yengo, L., Zheng, Z., et al.; eQTLGen Consortium (2018). Identifying gene targets for brain-related traits using transcriptomic and methylomic data from blood. Nat. Commun. *9*, 2282.
18. Skene, N.G., Bryois, J., Bakken, T.E., Breen, G., Crowley, J.J., Gaspar, H.A., Giusti-Rodriguez, P., Hodge, R.D., Miller, J.A., Muñoz-Manchado, A.B., et al.; Major Depressive Disorder Working Group of the Psychiatric Genomics Consortium (2018). Genetic identification of brain cell types underlying schizophrenia. Nat. Genet. *50*, 825–833.
19. Taylor, J.P., Brown, R.H., Jr., and Cleveland, D.W. (2016). Decoding ALS: from genes to mechanism. Nature *539*, 197–206.
20. Robins, C., Wingo, A.P., Fan, W., Duong, D.M., Meigs, J., Gerasimov, E.S., Dammer, E.B., Cutler, D.J., Jager, P.L.D., Bennett, D.A., et al. (2019). Genetic control of the human brain proteome. bioRxiv. https://doi.org/10.1101/816652.
21. Jansen, I.E., Savage, J.E., Watanabe, K., Bryois, J., Williams, D.M., Steinberg, S., Sealock, J., Karlsson, I.K., Hägg, S., Athanasiu, L., et al. (2019). Genome-wide meta-analysis identifies new loci and functional pathways influencing Alzheimer's disease risk. Nat. Genet. *51*, 404–413.
22. van Rheenen, W., Shatunov, A., Dekker, A.M., McLaughlin, R.L., Diekstra, F.P., Pulit, S.L., van der Spek, R.A., Võsa, U., de Jong, S., Robinson, M.R., et al.; PARALS Registry; SLALOM Group; SLAP Registry; FALS Sequencing Consortium; SLAGEN Consortium; and NNIPPS Study Group (2016). Genome-wide association analyses identify new risk variants and the genetic architecture of amyotrophic lateral sclerosis. Nat. Genet. *48*, 1043–1048.
23. Howard, D.M., Adams, M.J., Clarke, T.K., Hafferty, J.D., Gibson, J., Shirali, M., Coleman, J.R.I., Hagenaars, S.P., Ward, J., Wigmore, E.M., et al.; 23andMe Research Team; and Major Depressive Disorder Working Group of the Psychiatric Genomics Consortium (2019). Genome-wide meta-analysis of depression identifies 102 independent variants and highlights the importance of the prefrontal brain regions. Nat. Neurosci. *22*, 343–352.
24. Jansen, P.R., Watanabe, K., Stringer, S., Skene, N., Bryois, J., Hammerschlag, A.R., de Leeuw, C.A., Benjamins, J.S., Muñoz-Manchado, A.B., Nagel, M., et al.; 23andMe Research Team (2019). Genome-wide analysis of insomnia in 1,331,010 individuals identifies new risk loci and functional pathways. Nat. Genet. *51*, 394–403.
25. Savage, J.E., Jansen, P.R., Stringer, S., Watanabe, K., Bryois, J., de Leeuw, C.A., Nagel, M., Awasthi, S., Barr, P.B., Coleman, J.R.I., et al. (2018). Genome-wide association meta-analysis in 269,867 individuals identifies new genetic and functional links to intelligence. Nat. Genet. *50*, 912–919.
26. Elsworth, B.L., Mitchell, R., Raistrick, C.A., Paternoster, L., Hemani, G., and Gaunt, T. (2019). MRC IEU UK Biobank GWAS pipeline version 2. University of Bristol. https://doi.org/10.5523/bris.pnoat8cxo0u52p6ynfaekeigi.
27. Bipolar Disorder and Schizophrenia Working Group of the Psychiatric Genomics Consortium. Electronic address: douglas.ruderfer@vanderbilt.edu; and Bipolar Disorder and Schizophrenia Working Group of the Psychiatric Genomics Consortium (2018). Genomic Dissection of Bipolar Disorder and Schizophrenia, Including 28 Subphenotypes. Cell *173*, 1705–1715.e16.
28. Bennett, D.A., Buchman, A.S., Boyle, P.A., Barnes, L.L., Wilson, R.S., and Schneider, J.A. (2018). Religious Orders Study and Rush Memory and Aging Project. J. Alzheimers Dis. *64* (s1), S161–S189.
29. Karolchik, D., Hinrichs, A.S., Furey, T.S., Roskin, K.M., Sugnet, C.W., Haussler, D., and Kent, W.J. (2004). The UCSC Table Browser data retrieval tool. Nucleic Acids Res. *32*, D493–D496.

30. Chang, C.C., Chow, C.C., Tellier, L.C., Vattikuti, S., Purcell, S.M., and Lee, J.J. (2015). Second-generation PLINK: rising to the challenge of larger and richer datasets. Gigascience *4*, 7.
31. Bycroft, C., Freeman, C., Petkova, D., Band, G., Elliott, L.T., Sharp, K., Motyer, A., Vukcevic, D., Delaneau, O., O'Connell, J., et al. (2018). The UK Biobank resource with deep phenotyping and genomic data. Nature *562*, 203–209.
32. Sudlow, C., Gallacher, J., Allen, N., Beral, V., Burton, P., Danesh, J., Downey, P., Elliott, P., Green, J., Landray, M., et al. (2015). UK biobank: an open access resource for identifying the causes of a wide range of complex diseases of middle and old age. PLoS Med. *12*, e1001779.
33. De Jager, P.L., Ma, Y., McCabe, C., Xu, J., Vardarajan, B.N., Felsky, D., Klein, H.U., White, C.C., Peters, M.A., Lodgson, B., et al. (2018). A multi-omic atlas of the human frontal cortex for aging and Alzheimer's disease research. Sci. Data *5*, 180142.
34. Bowden, J., Del Greco M, F., Minelli, C., Zhao, Q., Lawlor, D.A., Sheehan, N.A., Thompson, J., and Davey Smith, G. (2019). Improving the accuracy of two-sample summary-data Mendelian randomization: moving beyond the NOME assumption. Int. J. Epidemiol. *48*, 728–742.
35. Bowden, J., Del Greco M, F., Minelli, C., Davey Smith, G., Sheehan, N.A., and Thompson, J.R. (2016). Assessing the suitability of summary data for two-sample Mendelian randomization analyses using MR-Egger regression: the role of the I2 statistic. Int. J. Epidemiol. *45*, 1961–1974.
36. Wald, A. (1940). The Fitting of Straight Lines if Both Variables are Subject to Error. Ann. Math. Stat. *11*, 284–300.
37. Hemani, G., Zheng, J., Elsworth, B., Wade, K.H., Haberland, V., Baird, D., Laurin, C., Burgess, S., Bowden, J., Langdon, R., et al. (2018). The MR-Base platform supports systematic causal inference across the human phenome. eLife *7*. e34408. https://doi.org/10.7554/eLife.34408.
38. Luciano, M., Hagenaars, S.P., Davies, G., Hill, W.D., Clarke, T.K., Shirali, M., Harris, S.E., Marioni, R.E., Liewald, D.C., Fawns-Ritchie, C., et al. (2018). Association analysis in over 329,000 individuals identifies 116 independent variants influencing neuroticism. Nat. Genet. *50*, 6–11.
39. Giambartolomei, C., Vukcevic, D., Schadt, E.E., Franke, L., Hingorani, A.D., Wallace, C., and Plagnol, V. (2014). Bayesian test for colocalisation between pairs of genetic association studies using summary statistics. PLoS Genet. *10*, e1004383.
40. Hormozdiari, F., van de Bunt, M., Segrè, A.V., Li, X., Joo, J.W.J., Bilow, M., Sul, J.H., Sankararaman, S., Pasaniuc, B., and Eskin, E. (2016). Colocalization of GWAS and eQTL Signals Detects Target Genes. Am. J. Hum. Genet. *99*, 1245–1260.
41. Richardson, T.G., Haycock, P.C., Zheng, J., Timpson, N.J., Gaunt, T.R., Davey Smith, G., Relton, C.L., and Hemani, G. (2018). Systematic Mendelian randomization framework elucidates hundreds of CpG sites which may mediate the influence of genetic variants on disease. Hum. Mol. Genet. *27*, 3293–3304.
42. Geisler, S., Huang, S.X., Strickland, A., Doan, R.A., Summers, D.W., Mao, X., Park, J., DiAntonio, A., and Milbrandt, J. (2019). Gene therapy targeting SARM1 blocks pathological axon degeneration in mice. J. Exp. Med. *216*, 294–303.
43. Gotoda, T. (2015). From Association to Function in the Post-GWAS Era. J. Atheroscler. Thromb. *22*, 442–444.
44. Hatcher, C., Relton, C.L., Gaunt, T.R., and Richardson, T.G. (2019). Leveraging brain cortex-derived molecular data to elucidate epigenetic and transcriptomic drivers of complex traits and disease. Transl. Psychiatry *9*, 105.
45. Vosberg, D.E., Leyton, M., and Flores, C. (2019). The Netrin-1/DCC guidance system: dopamine pathway maturation and psychiatric disorders emerging in adolescence. Mol. Psychiatry. *25*, 297–307.
46. Li, J., Ma, Z., Shi, M., Malty, R.H., Aoki, H., Minic, Z., Phanse, S., Jin, K., Wall, D.P., Zhang, Z., et al. (2015). Identification of Human Neuronal Protein Complexes Reveals Biochemical Activities and Convergent Mechanisms of Action in Autism Spectrum Disorders. Cell Syst. *1*, 361–374.
47. Thibaudeau, T.A., Anderson, R.T., and Smith, D.M. (2018). A common mechanism of proteasome impairment by neurodegenerative disease-associated oligomers. Nat. Commun. *9*, 1097.
48. Dantuma, N.P., and Bott, L.C. (2014). The ubiquitin-proteasome system in neurodegenerative diseases: precipitating factor, yet part of the solution. Front. Mol. Neurosci. *7*, 70.
49. Geisler, S., Doan, R.A., Strickland, A., Huang, X., Milbrandt, J., and DiAntonio, A. (2016). Prevention of vincristine-induced peripheral neuropathy by genetic deletion of SARM1 in mice. Brain *139*, 3092–3108.
50. Henninger, N., Bouley, J., Sikoglu, E.M., An, J., Moore, C.M., King, J.A., Bowser, R., Freeman, M.R., and Brown, R.H., Jr. (2016). Attenuated traumatic axonal injury and improved functional outcome after traumatic brain injury in mice lacking Sarm1. Brain *139*, 1094–1105.
51. Zhang, B., Gaiteri, C., Bodea, L.G., Wang, Z., McElwee, J., Podtelezhnikov, A.A., Zhang, C., Xie, T., Tran, L., Dobrin, R., et al. (2013). Integrated systems approach identifies genetic nodes and networks in late-onset Alzheimer's disease. Cell *153*, 707–720.
52. GTEx Consortium. (2017). Genetic effects on gene expression across human tissues. Nature *550*, 204–213.
53. Waddington, C.H. (1959). Canalization of development and genetic assimilation of acquired characters. Nature *183*, 1654–1655.
54. Siegal, M.L., and Bergman, A. (2002). Waddington's canalization revisited: developmental stability and evolution. Proc. Natl. Acad. Sci. USA *99*, 10528–10532.

The American Journal of Human Genetics, Volume *106*

Supplemental Data

Characterizing the Causal Pathway for Genetic Variants Associated with Neurological Phenotypes Using Human Brain-Derived Proteome Data

Nelson K. Kibinge, Caroline L. Relton, Tom R. Gaunt, and Tom G. Richardson

Supplementary Figure 1 – A flowchart of the analysis pipeline for this study and key results

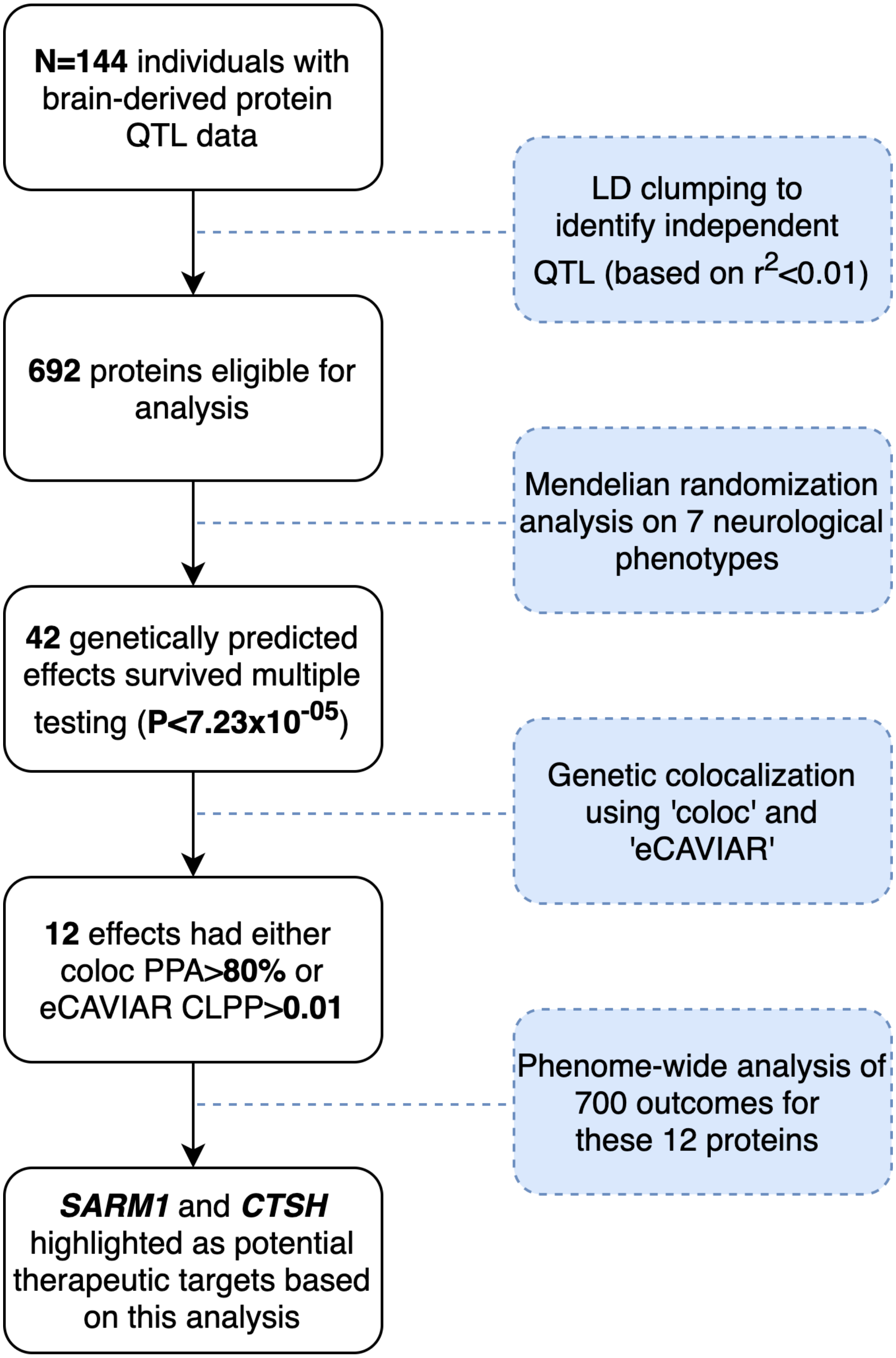

Supplementary Figure 2 – Gene expression profile for *ABCB9* across 54 tissue types in the GTEx project

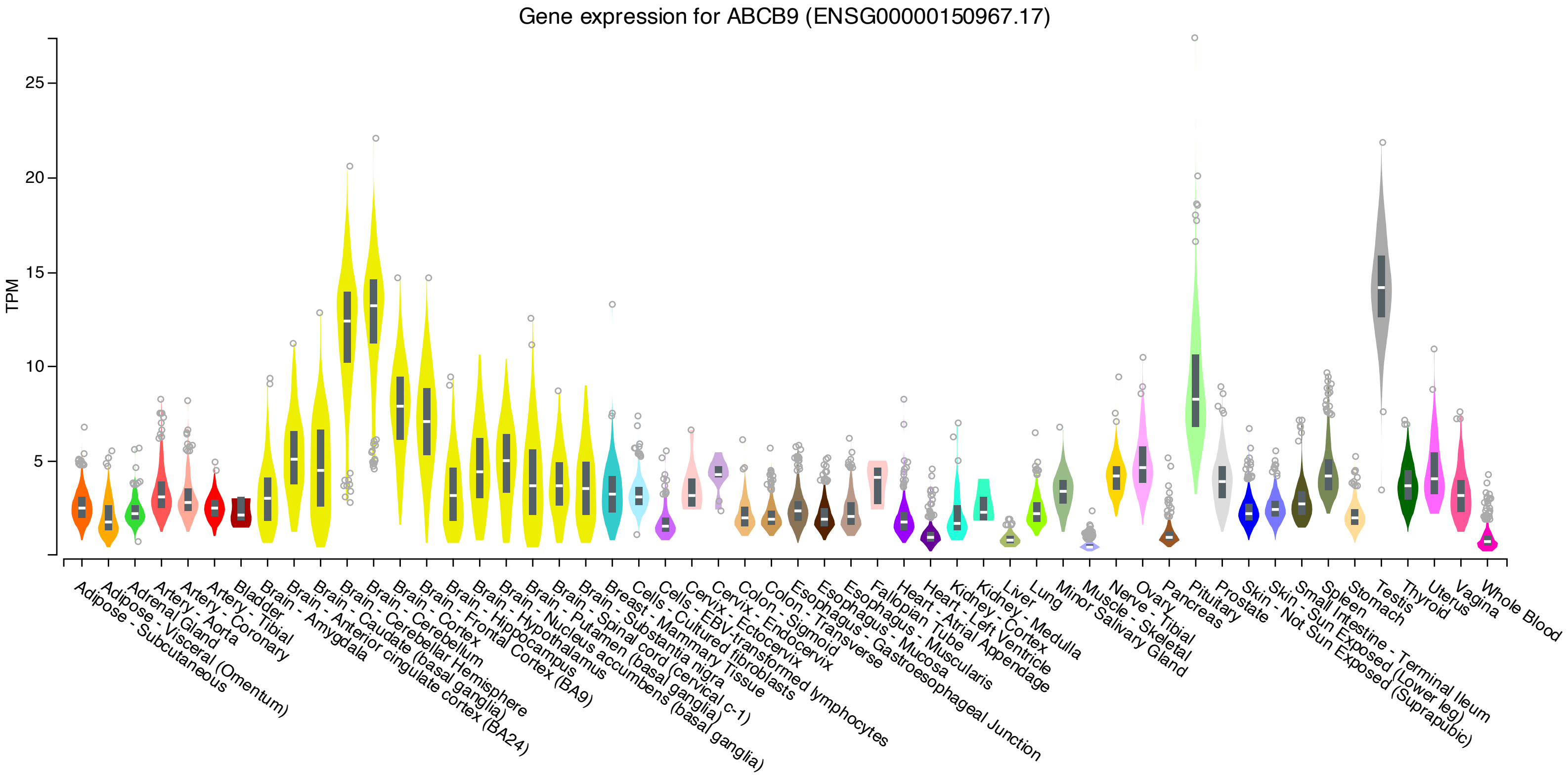

Supplementary Figure 3 – Gene expression profile for *CTSH* across 54 tissue types in the GTEx project

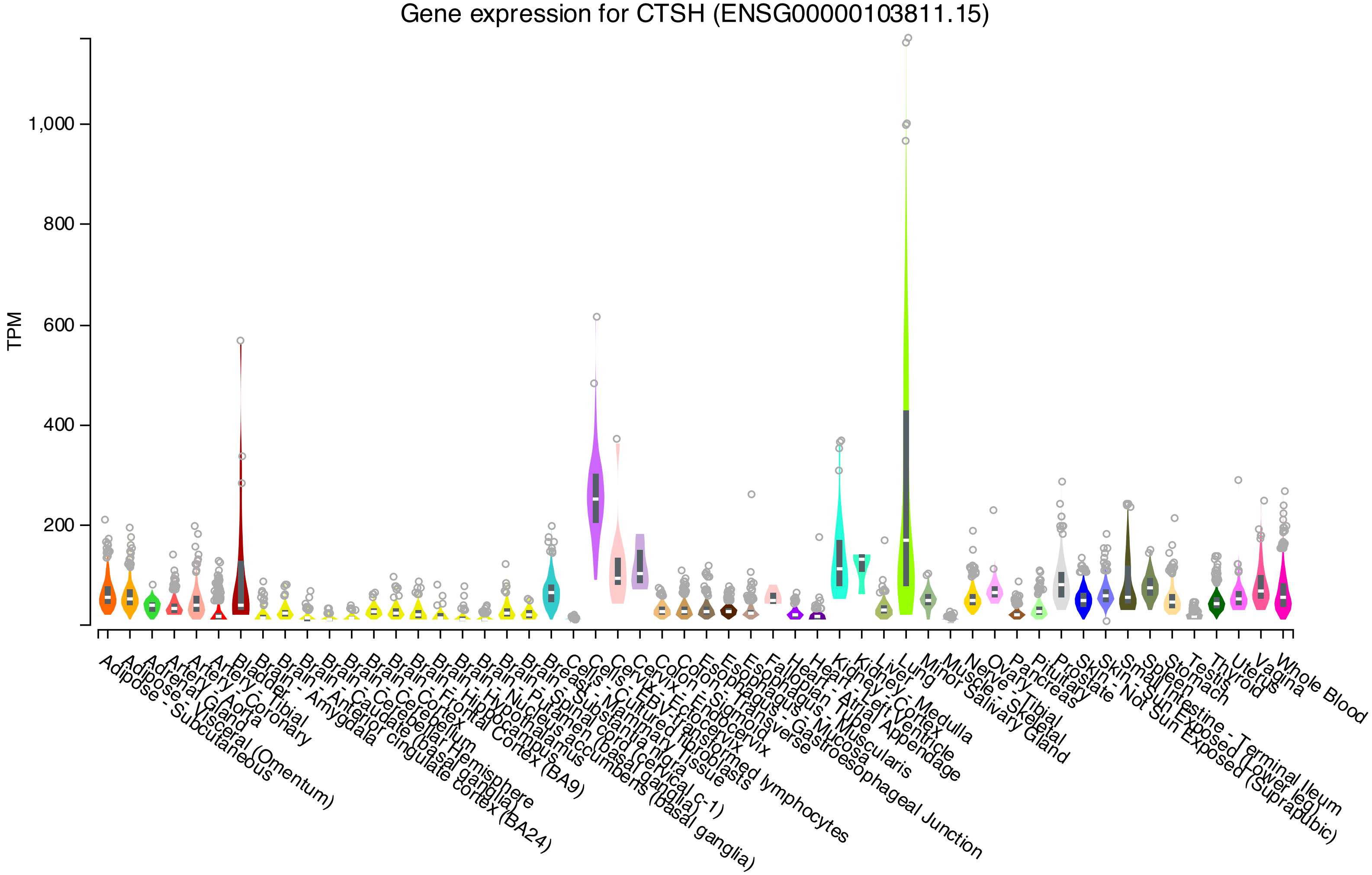

Supplementary Figure 4 – Gene expression profile for *DCC* across 54 tissue types in the GTEx project

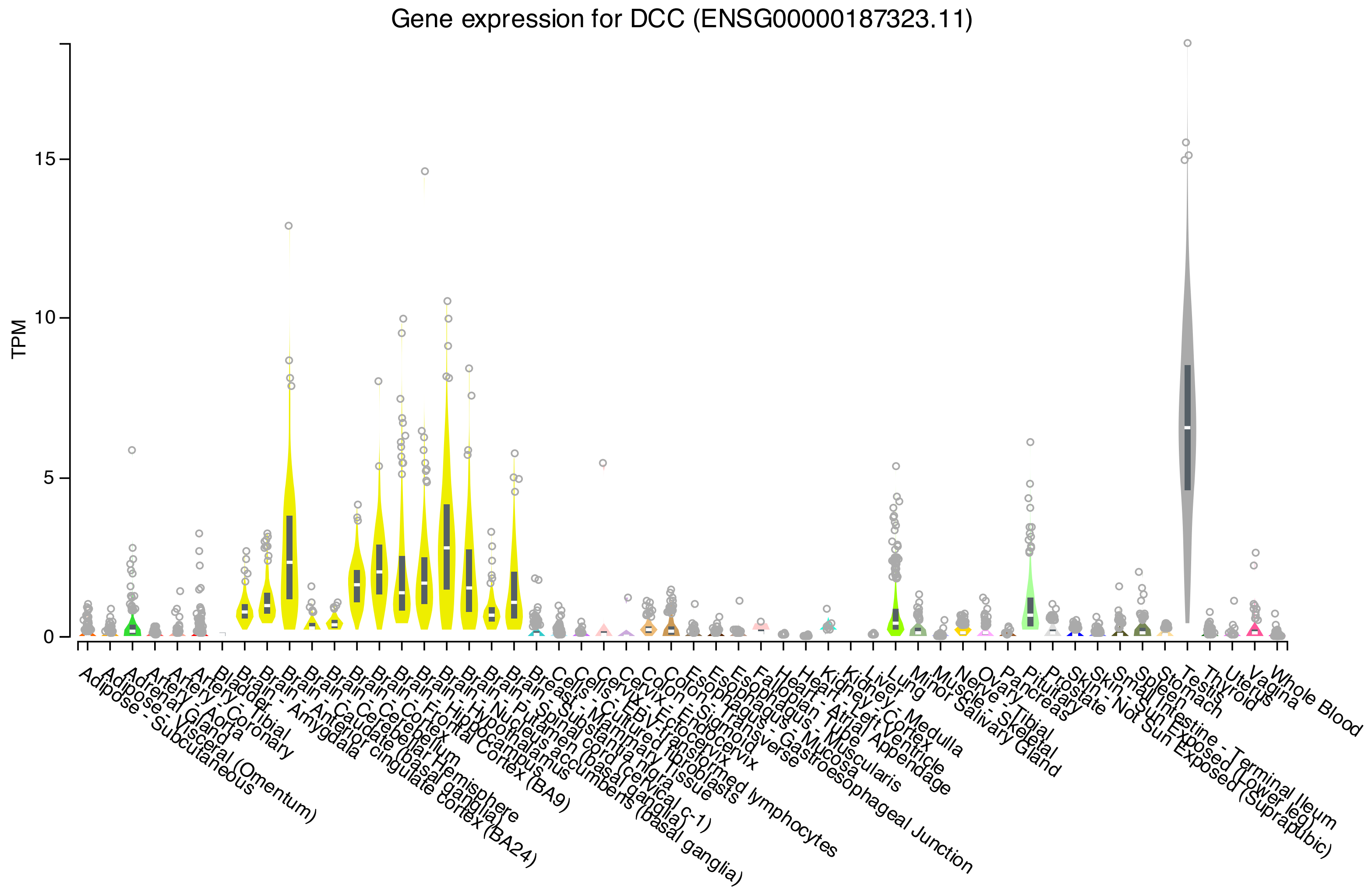

Supplementary Figure 5– Gene expression profile for *ERLIN1* across 54 tissue types in the GTEx project

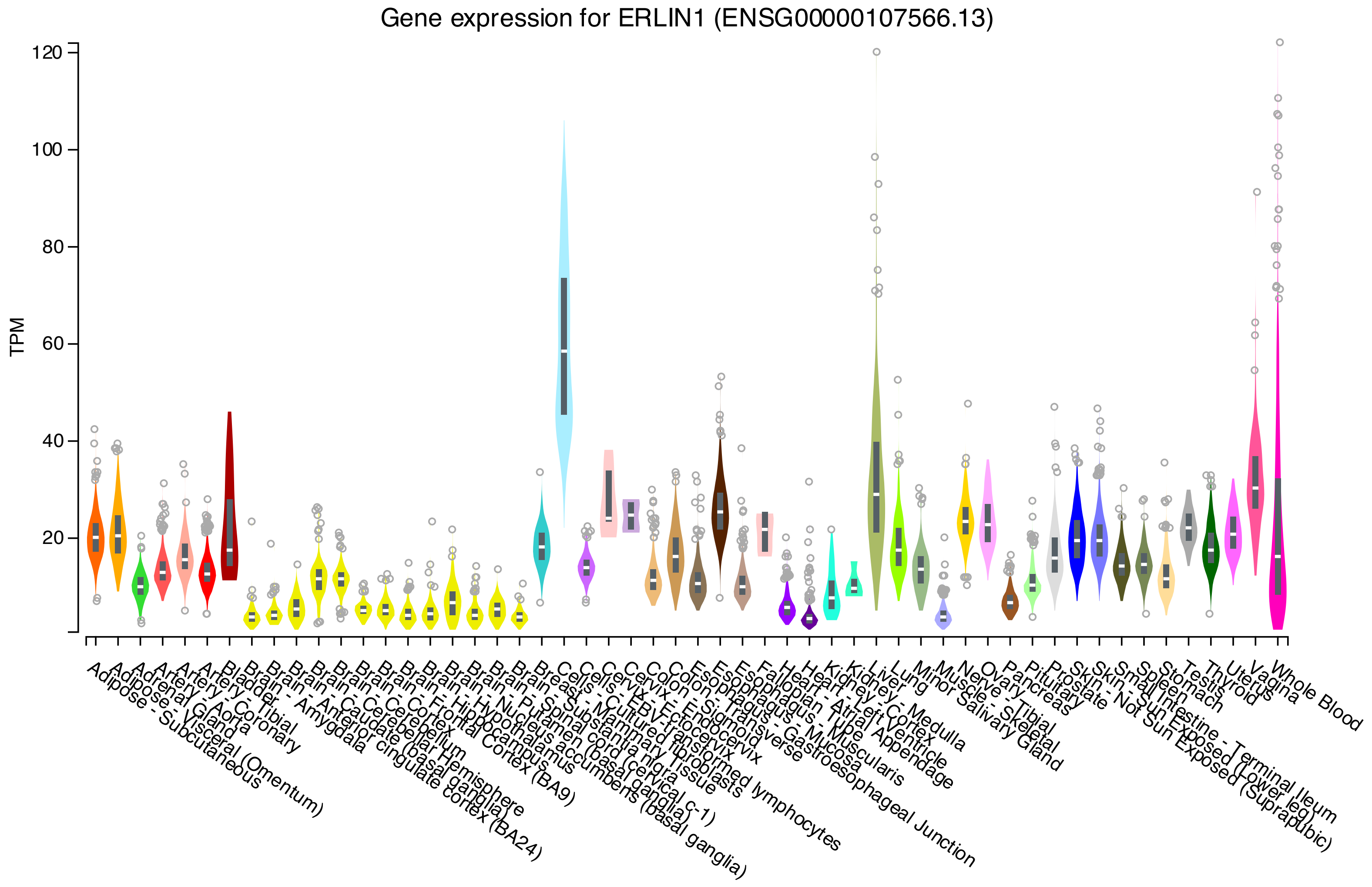

Supplementary Figure 6 – Gene expression profile for *FLOT2* across 54 tissue types in the GTEx project

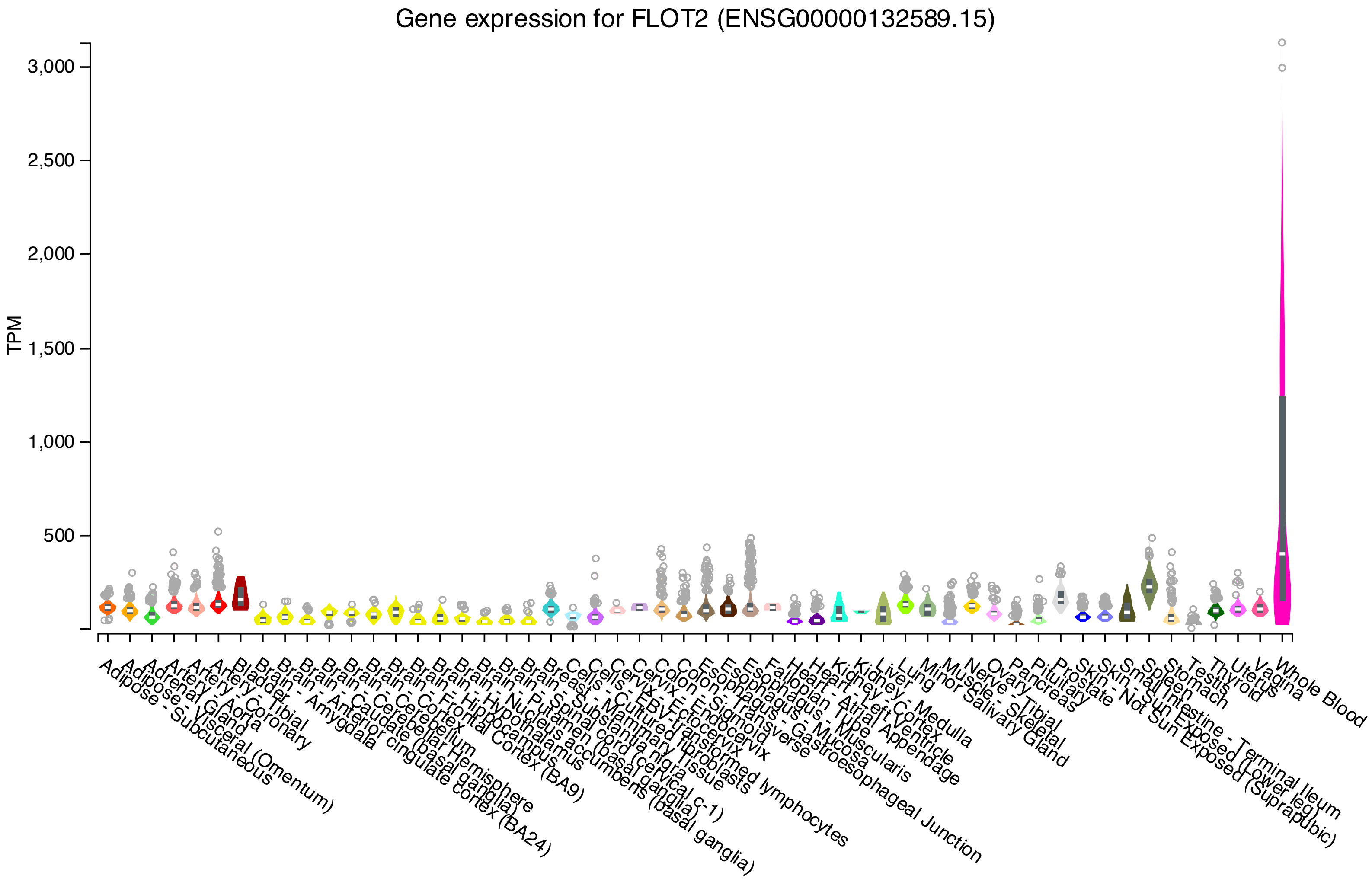

Supplementary Figure 7 – Gene expression profile for *KHK* across 54 tissue types in the GTEx project

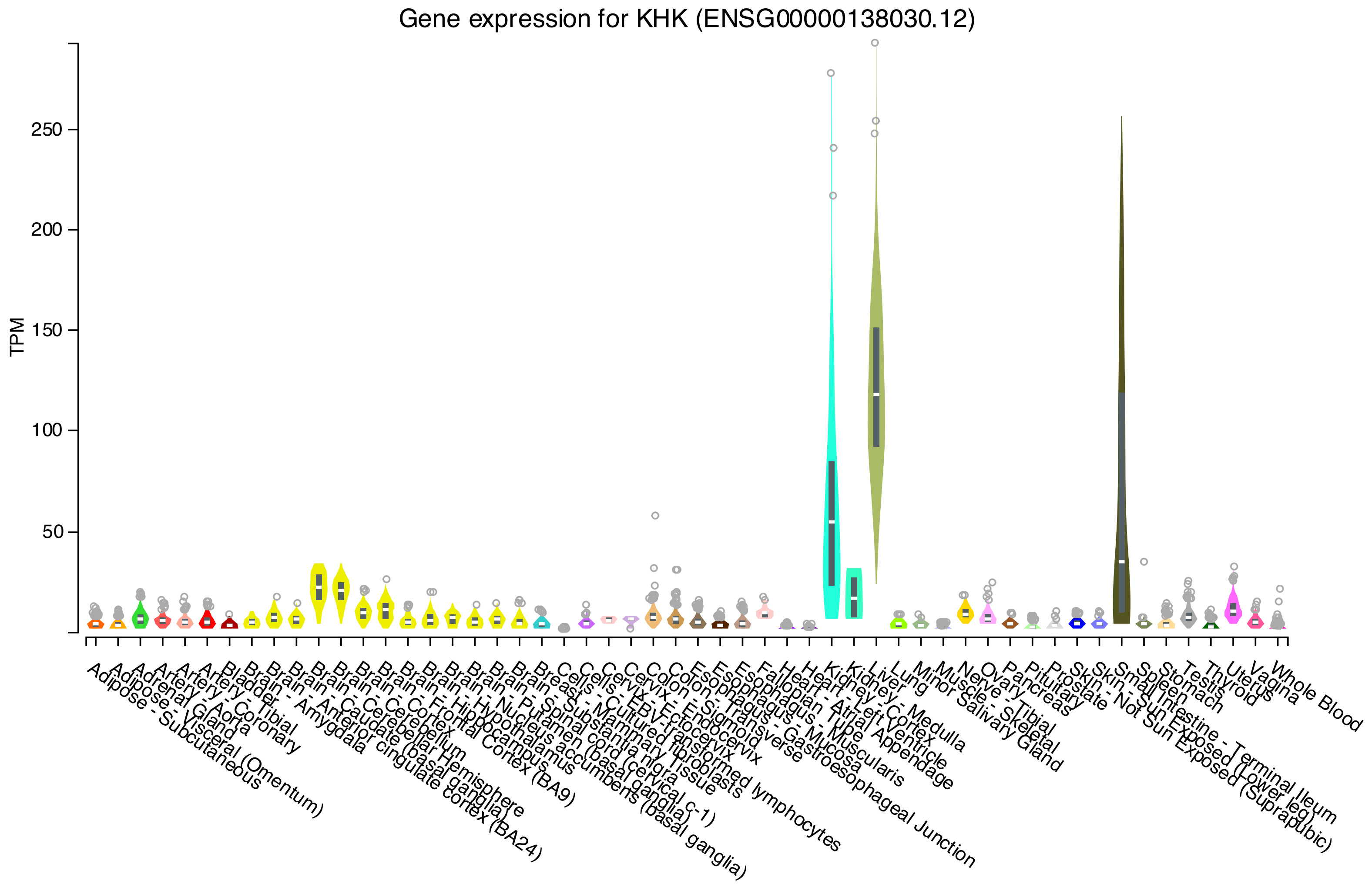

Supplementary Figure 8 – Gene expression profile for *PANK4* across 54 tissue types in the GTEx project

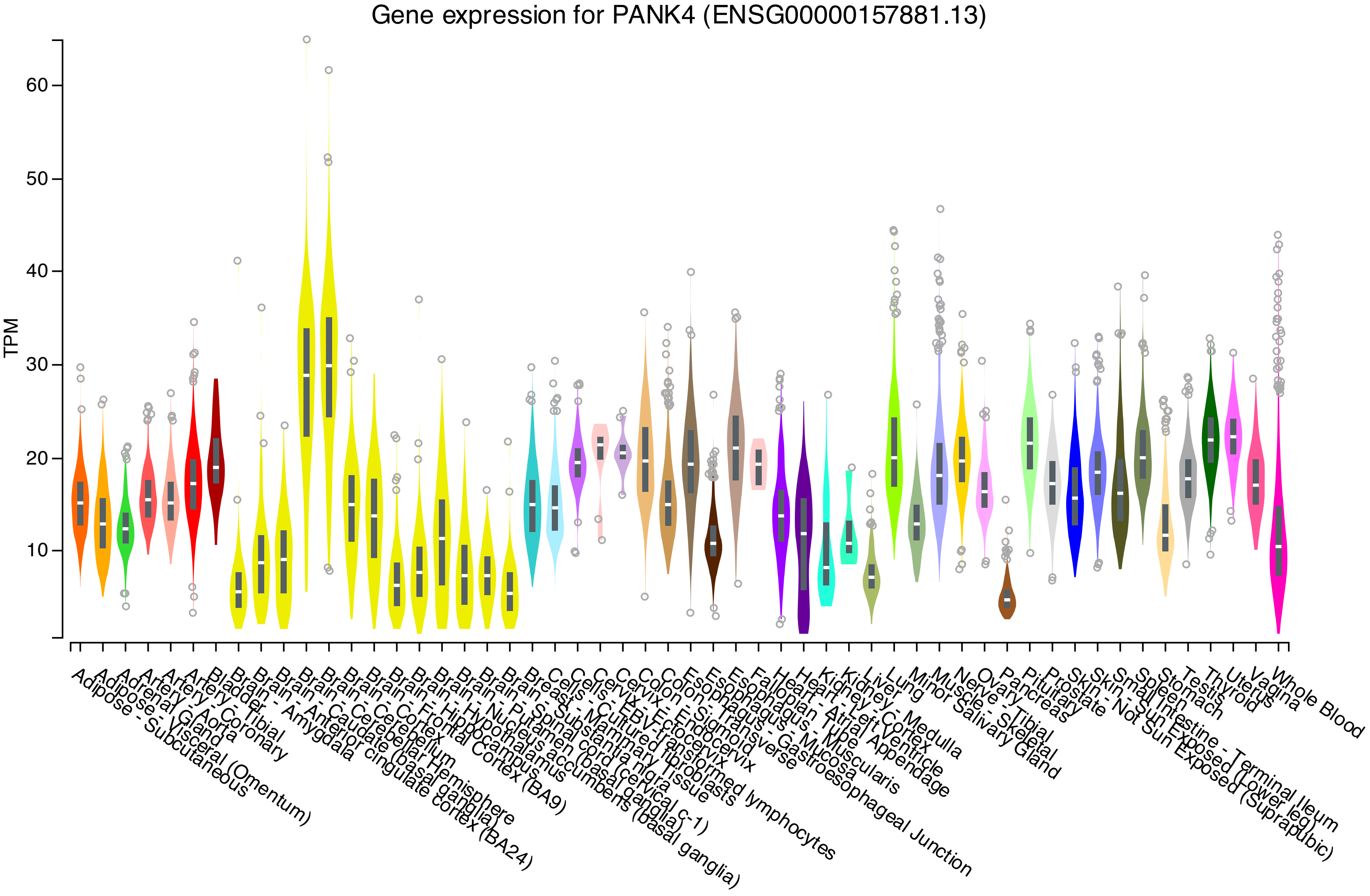

Supplementary Figure 9 – Gene expression profile for *PSMB4* across 54 tissue types in the GTEx project

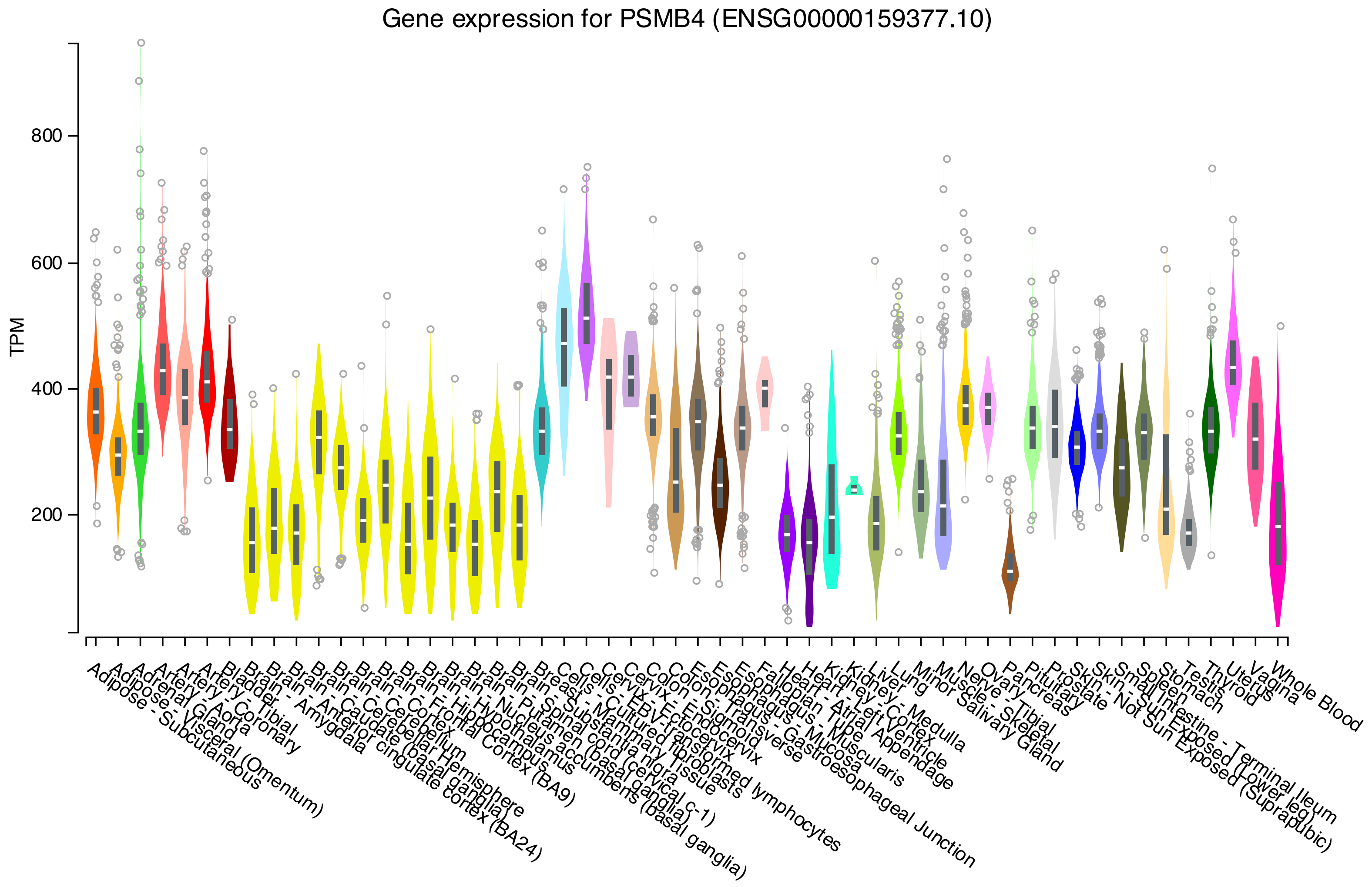

Supplementary Figure 10 – Gene expression profile for *RLBP1* across 54 tissue types in the GTEx project

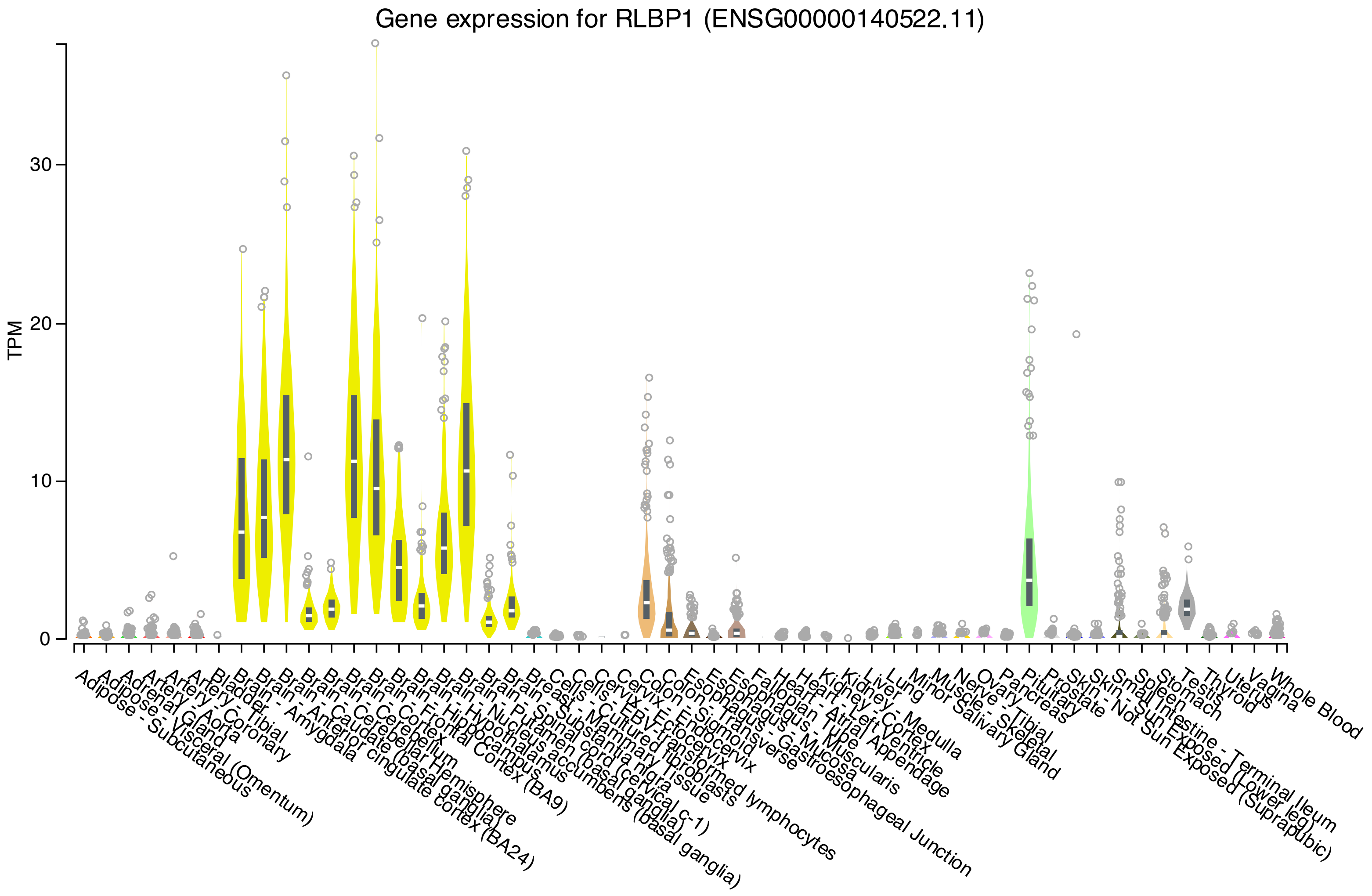

Supplementary Figure 11 – Gene expression profile for *SARM1* across 54 tissue types in the GTEx project

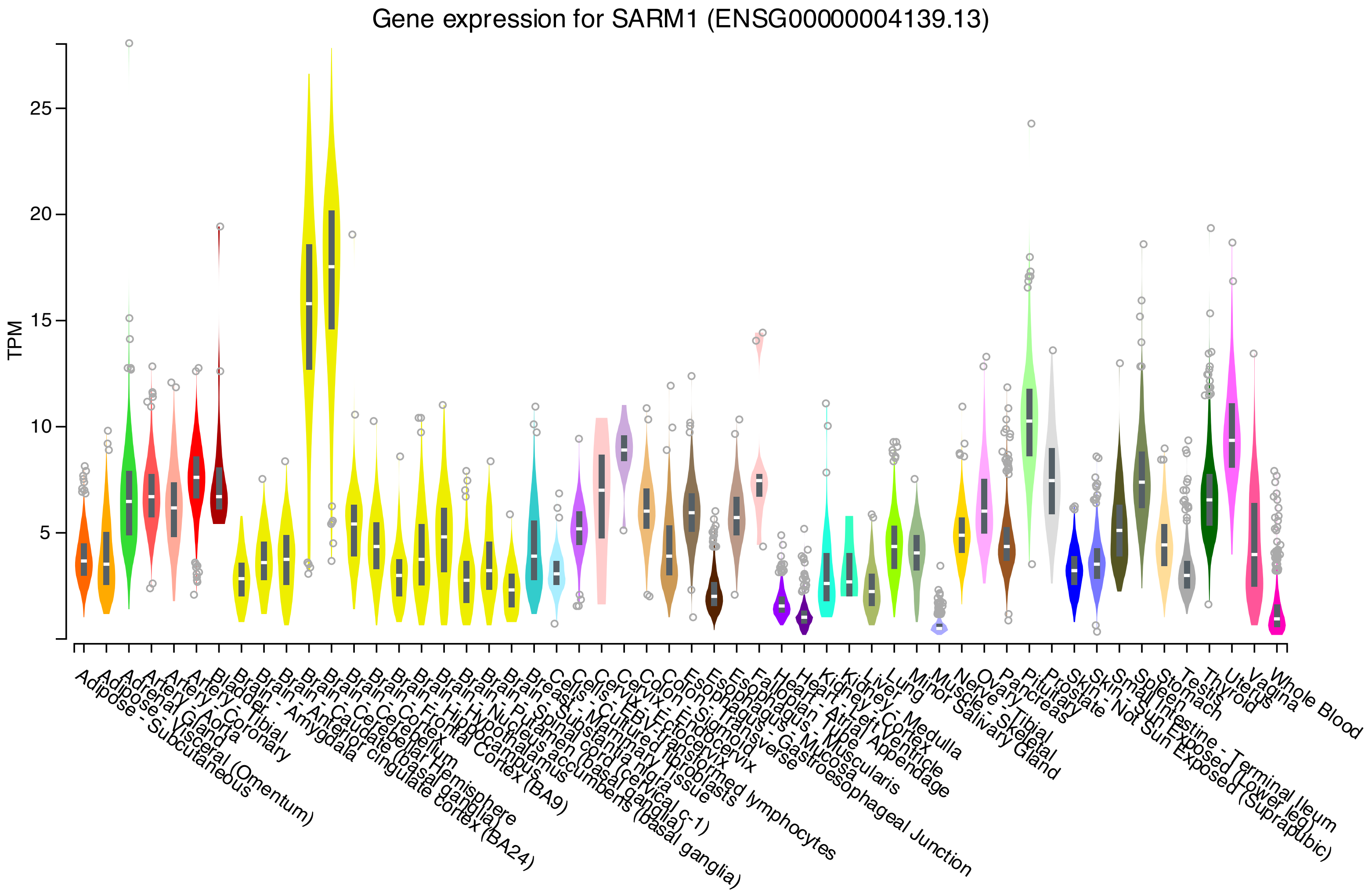

Supplementary Figure 12 – Gene expression profile for *SIDT1* across 54 tissue types in the GTEx project

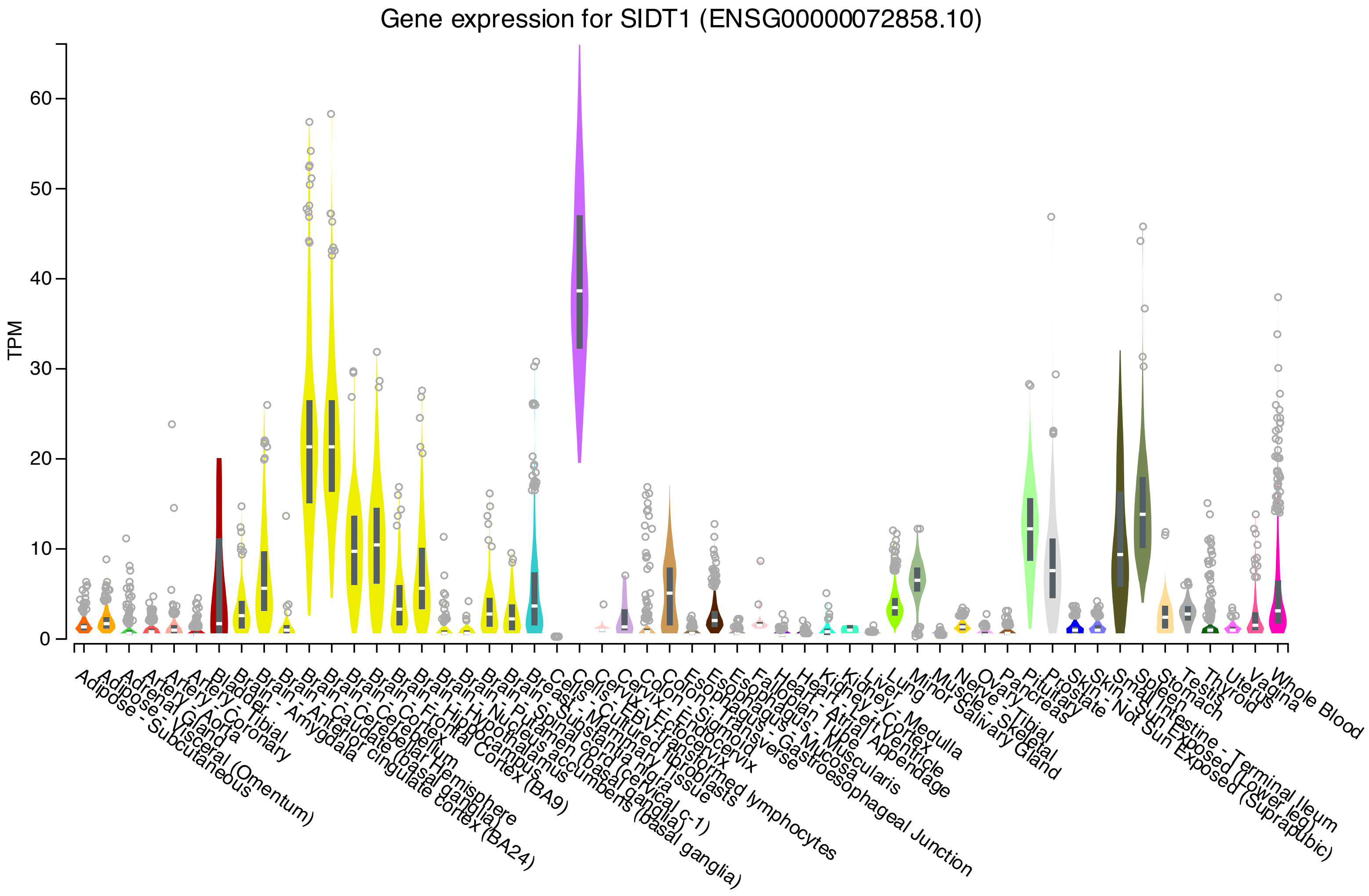

Supplementary Figure 13 – Gene expression profile for *SNX32* across 54 tissue types in the GTEx project

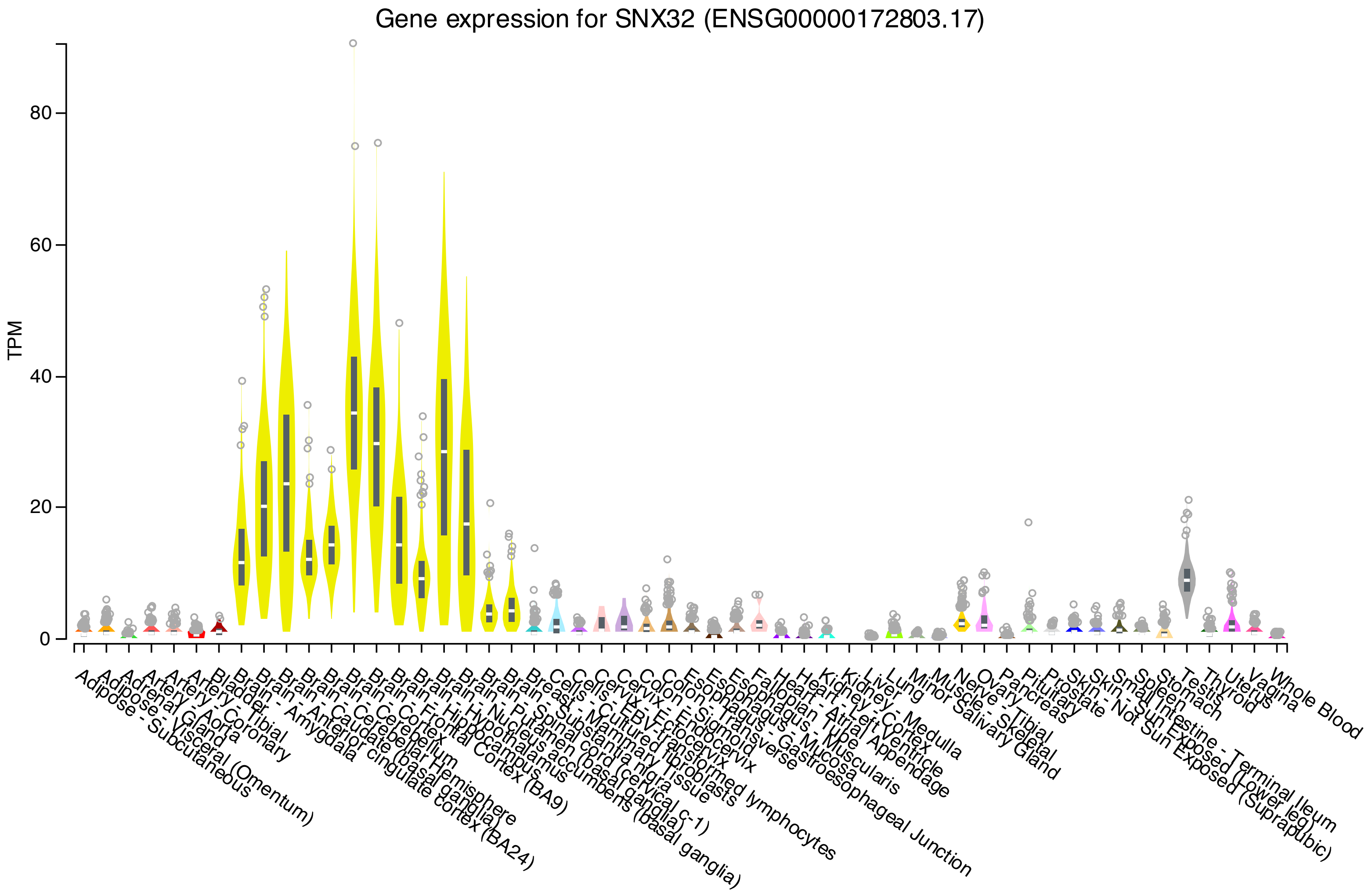

Supplementary Table 1– Characteristics of genome-wide association studies of neurological and psychiatric traits

Trait/disease	Author	Year	URL
Alzheimer's disease	Jansen et al	2019	https://ctg.cncr.nl/software/summary_statistics
Amyotrophic lateral sclerosis	van Rheenen et al	2016	http://databrowser.projectmine.com/
Depression	Howard et al	2019	https://datashare.is.ed.ac.uk/handle/10283/3203
Insomnia	Jansen et al	2018	https://ctg.cncr.nl/software/summary_statistics
Intelligence	Savage et al	2018	https://ctg.cncr.nl/software/summary_statistics
Neuroticism	Elsworth et al [unpublished]	2018	mrbase.org
Schizophrenia	Ruderfer et al	2018	https://www.med.unc.edu/pgc/results-and-downloads
